# CDKAL1 Drives the Maintenance of Cancer Stem‐Like Cells by Assembling the eIF4F Translation Initiation Complex

**DOI:** 10.1002/advs.202206542

**Published:** 2023-02-14

**Authors:** Rongsheng Huang, Takahiro Yamamoto, Eiji Nakata, Toshifumi Ozaki, Kazuhiko Kurozumi, Fanyan Wei, Kazuhito Tomizawa, Atsushi Fujimura

**Affiliations:** ^1^ Department of Cellular Physiology Okayama University Graduate School of Medicine, Dentistry, and Pharmaceutical Sciences Okayama Okayama 700‐8558 Japan; ^2^ Department of Molecular Physiology Kumamoto University Faculty of Life Sciences Kumamoto Kumamoto 860‐0811 Japan; ^3^ Department of Orthopedic Surgery Okayama University Graduate School of Medicine, Dentistry, and Pharmaceutical Sciences Okayama Okayama 700‐8558 Japan; ^4^ Department of Neurosurgery Hamamatsu University School of Medicine Hamamatsu Shizuoka 431‐3192 Japan; ^5^ Department of Modomics Biology and Medicine Institute of Development, Aging and Cancer Tohoku University Sendai Miyagi 980‐8575 Japan; ^6^ Neutron Therapy Research Center Okayama University Okayama Okayama 700‐8558 Japan

**Keywords:** cancer stem‐like cells, CG‐rich 5’UTR, eIF4F complex, CDKAL1, SALL2

## Abstract

Cancer stem‐like cells (CSCs) have a unique translation mode, but little is understood about the process of elongation, especially the contribution of tRNA modifications to the maintenance of CSCs properties. Here, it is reported that, contrary to the initial aim, a tRNA‐modifying methylthiotransferase CDKAL1 promotes CSC‐factor SALL2 synthesis by assembling the eIF4F translation initiation complex. CDKAL1 expression is upregulated in patients with worse prognoses and is essential for maintaining CSCs in rhabdomyosarcoma (RMS) and common cancers. Translatome analysis reveals that a group of mRNAs whose translation is CDKAL1‐dependent contains cytosine‐rich sequences in the 5’ untranslated region (5’UTR). Mechanistically, CDKAL1 promotes the translation of such mRNAs by organizing the eIF4F translation initiation complex. This complex formation does not require the enzyme activity of CDKAL1 but requires only the NH_2_‐terminus domain of CDKAL1. Furthermore, sites in CDKAL1 essential for forming the eIF4F complex are identified and discovered candidate inhibitors of CDKAL1‐dependent translation.

## Graphical abstract

It is reported that a tRNA‐modifying methylthiotransferase CDKAL1 promotes cancer stem‐like cells (CSC)‐factor SALL2 synthesis by assembling the eIF4F translation initiation complex. CDKAL1 expression is upregulated in patients with worse prognoses and is essential for maintaining CSCs in rhabdomyosarcoma (RMS) and common cancers. Translatome analysis reveals that a group of mRNAs whose translation is CDKAL1‐dependent contains cytosine‐rich sequences in the 5’ untranslated region (5’UTR).

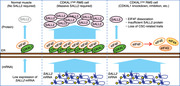

## 1 Introduction

Protein synthesis, a two‐step process of transcription and translation, is a fundamental determinant of cancer phenotype.^[^ ^1^, ^2^ ^]^ In search of the determinants of cancer stem‐like cells (CSCs) properties, much effort has been expended on the studies to identify transcription factors and cofactors that confer CSC‐related traits on cancer cells and the mechanisms underlying transcriptional modal shifts such as epigenetic alteration.^[^ ^3^, ^4^, ^5^, ^6^ ^]^ The findings greatly explain the unique gene expression and proteome profiles of CSCs that result in phenotypic differences between CSCs and non‐CSCs. On the other hand, while there is growing evidence that cancer cells have a distinct translation mode from normal cells^[^ ^7^, ^8^, ^9^, ^10^ ^]^ and that CSCs have unique mechanisms by which alter the translation efficacy such as circular RNAs and long noncoding RNAs,^[^ ^11^, ^12^ ^]^ it is not fully understood how differences in the translational phase account for the formation and maintenance of CSCs.

The translation process consists of three phases: translation initiation, elongation, and termination. Recent advances in translatome techniques have unveiled a discrepancy between transcriptome and translatome in cancer cells.^[^ ^10^, ^13^, ^14^ ^]^ It has been revealed that, during tumorigenesis, the expression levels of CSC‐related factors are elevated through a translation initiation regulation.^[^ ^10^ ^]^ In the initiation phase, oncogenic signaling pathways control eukaryotic initiation factors (eIFs), whose abnormal activities result in a selective translation of mRNAs that confer tumor‐initiating capacity and therapeutic resistance on cancer cells and thus contributed to cancer expansion.^[^ ^9^, ^15^ ^]^ In contrast to the initiation phase, the contribution of the elongation phase and the termination phase to the maintenance of CSC properties is less understood. At an elongation phase, tRNA plays a pivotal role in controlling the rate of nascent protein synthesis as it carries a corresponding amino acid to the ribosome. The corresponding enzymes modify nucleotides in tRNAs in various ways at various sites.^[^ ^16^, ^17^ ^]^ It is believed that these modifications contribute to efficient and accurate elongation by increasing codon–anticodon stability or the stability of the tRNA itself, and there is increasing evidence to suggest that they are involved in CSCs maintenance.^[^ ^18^, ^19^, ^20^ ^]^ In addition to directly affecting the elongation process, a tRNA modification enzyme also contributes to CSCs maintenance by protecting them from cytotoxic modified nucleotides, as we have previously reported.^[^ ^21^ ^]^ However, whether tRNA modification impacts the elongation phase in the translation of specific mRNAs that determine CSC characteristics remains unclear.

We initiated this study to identify the tRNA‐modifying enzymes defining the CSC properties in rhabdomyosarcoma (RMS), where protein synthesis is much less understood than in other cancers. Rhabdomyosarcoma (RMS) is a rare but most common malignant soft tissue tumor in young children.^[^ ^22^ ^]^ Although recent advances in integrative and comprehensive treatment have improved patient survival, the prognosis of patients with metastases or recurrence remains poor, probably due to the CSC population.^[^ ^23^, ^24^, ^25^ ^]^ Here, we find that the expression levels of CDKAL1, a member of the methylthiotransferase family that has been associated with susceptibility to type II diabetes,^[^ ^26^, ^27^, ^28^, ^29^ ^]^ are significantly elevated in a CSC population of RMS cells. CDKAL1 is required to maintain the CSC‐related traits in RMS, as well as common cancers. Because CDKAL1 specifically converts N^6^‐threonylcarbamoyl adenosine (t^6^A) to 2‐methylthio‐N^6^‐threonylcarbamoyl adenosine (ms^2^t^6^A) in the cytosolic tRNA^Lys^ (UUU),^[^ ^29^ ^]^ we expected that CDKAL1 impacted the elongation efficacy, especially of mRNAs that have a high content of AAA/AAG lysine codons. However, unexpectedly, we reveal that CDKAL1 is essential for assembling the eIF4F translation initiation complex and for promoting the efficient translation of the CSC‐related transcriptional factor SALL2. We confirm that this mechanism is not limited to CSCs maintenance in RMS but is universally applicable to CSCs in common cancers, such as melanoma, liver cancer, prostate cancer, stomach cancer, and glioma.

## 2 Results

### 2.1 CDKAL1 Is Highly Expressed in RMS CSCs

**Figure 1:**
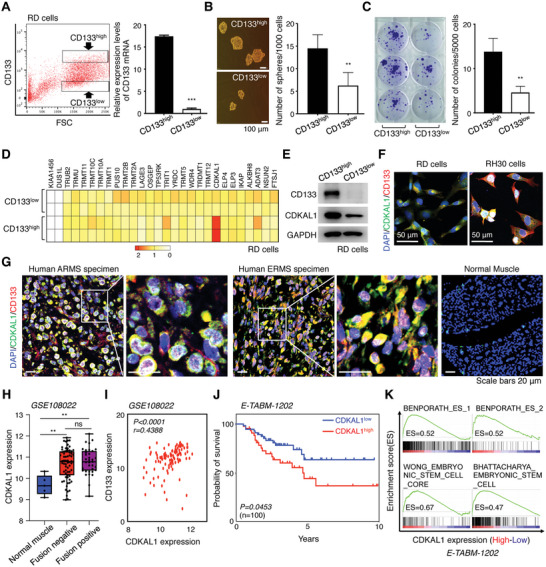
CDKAL1 is highly expressed in rhabdomyosarcoma (RMS) cancer stem‐like cells (CSCs). A) The human RMS cell line RD was fractionated by FACS according to CD133 expression level. The level in each fraction was confirmed by quantitative PCR (*n* = 2, error bars indicate mean ± SD). B,C) The self‐renewal (B, *n = 4*) and colony‐forming (C, *n* = 3) capacities of CD133^high^ and CD133^low^ RD cells (error bars indicate mean ± SD). D) Quantitative PCR for the expression profiling of tRNA‐modifying enzymes was performed in the CD133^high^ and CD133^low^ RD cells. (E) Western blotting analysis to validate the elevated expression level of CDKAL1 in the CD133^high^ RD cells. Note that this population harbored higher CSC‐related potential than the CD133^low^ RD cell population (Figure 1B,C). F) Immunofluorescent analysis using anti‐CDKAL1 and anti‐CD133 antibodies in RD (left) and RH30 (right) cells. Scale bars, 50 µm. G) Immunofluorescence analysis of normal skeletal muscle and pathological specimens of alveolar RMS (ARMS) and embryonal RMS (ERMS) using anti‐CDKAL1 and anti‐CD133 antibodies. Scale bars, 20 µm. H) Comparison of CDKAL1 expression in patients with fusion‐negative and fusion‐positive RMS in the GSE108022 dataset. I) The positive correlation between the expression levels of CD133 and those of CDKAL1 in a gene set of RMS patients (GSE108022). J) Kaplan‐Meier curve displaying the prognosis in RMS patients with CDKAL1^high^ and CDKAL1^low^ signature from the E‐TABM‐1202 dataset. K) Gene set enrichment analysis (GSEA) displaying the stem cell‐related gene sets that are enriched in the CDKAL1^high^ RMS population in the E‐TABM‐1202 dataset.

To compare the gene expression profile of tRNA‐modifying enzymes in CSCs and non‐CSCs, we isolated CD133^high^ and CD133^low^ populations from the human RMS cell line RD by fluorescence‐activated cell sorting (FACS) (**Figure** **1**A) because CD133 is a well‐known CSC marker and can be used to isolate CSCs from RD cells.^[^ ^23^, ^24^ ^]^ CD133^high^ RD cells have higher self‐renewal and colony‐forming capacities than CD133^low^ RD cells (Figure 1B,C), indicating that we successfully isolated CSCs. We selected 28 tRNA‐modifying enzymes whose nucleic acid modification mode and target tRNAs have been defined^[^ ^30^ ^]^ and quantified their expression levels in each population. We identified *CDKAL1* as a highly expressed gene in the CD133^high^ population compared to the CD133^low^ population in RD cells (Figure 1D,E). RMS consists of two major histological subtypes, embryonal (ERMS) and alveolar (ARMS), or two major genetic subtypes, “fusion‐negative” and “fusion‐positive” for *PAX3‐FOXO1A* or *PAX7‐FOXO1A* genes.^[^ ^31^ ^]^ CDKAL1 enrichment in the CD133^high^ population was observed not only in RD (ERMS, fusion‐negative) but also in RH30 (ARMS, fusion‐positive) cells (Figure 1F). Immunostaining analysis showed that CDKAL1 was also highly expressed in specimens from ARMS and ERMS patients (Figure 1G).

To examine whether the CDKAL1 expression level affects RMS progression, we analyzed the GSE108022 gene expression dataset.^[^ ^31^ ^]^ We found that CDKAL1 was expressed at higher levels in both fusion‐negative and fusion‐positive RMS than normal skeletal muscle tissue (Figure 1H). We also found a positive correlation between the expression levels of CDKAL1 and CD133, suggesting that CDKAL1^high^ RMS harbors CSC‐related traits (Figure 1I). We next analyzed the E‐TABM‐1202 dataset, which contains gene expression profiles and clinical information for each patient.^[^ ^32^ ^]^ We found that the prognosis in CDKAL1^high^ patients was significantly worse than in the CDKAL1^low^ patients (Figure 1J). We then performed gene set enrichment analysis (GSEA) on the same dataset and discovered that, in RMS patients, *CDKAL1* expression was positively correlated with the expression of genes associated with stem cell properties (Figure 1K). These findings suggest that CDKAL1 determines RMS progression by affecting the CSC properties.

### 2.2 CDKAL1 Promotes the Maintenance of CSC‐Related Traits in RMS as Well as Other Types of Cancer

**Figure 2:**
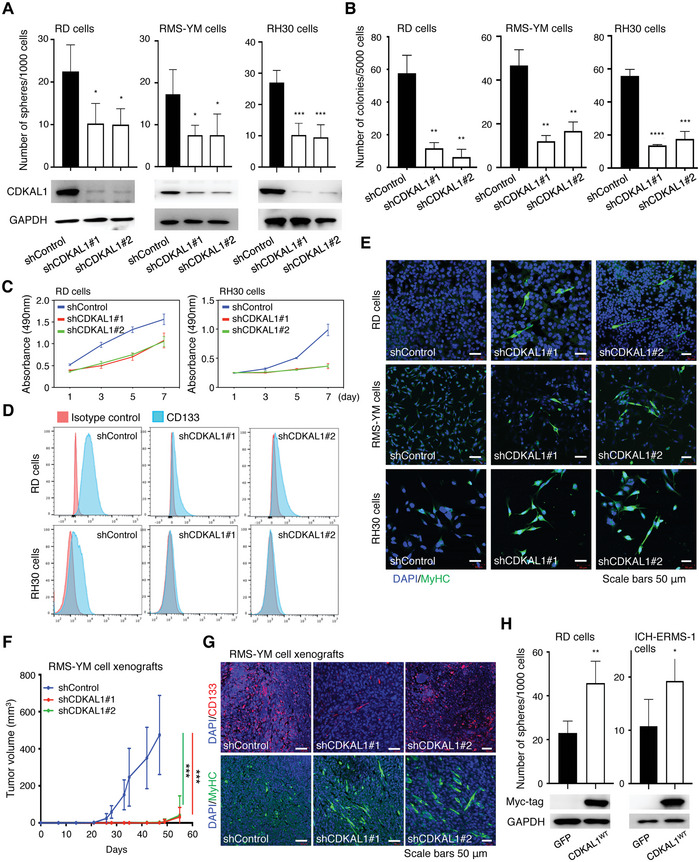
CDKAL1 promotes the maintenance of CSC‐related traits in RMS. A) The self‐renewal capacity of Control‐shRNA‐ or *CDKAL1*‐shRNA‐expressing RMS cell lines was measured (*n* = 4, error bars indicate mean ± SD). Western blotting results are shown to confirm the *CDKAL1* knockdown. B) The clonogenic growth potential of Control‐shRNA‐ or *CDKAL1*‐shRNA‐expressing RMS cells (*n* = 3, error bars indicate mean ± SD). C) The cell proliferation analysis of Control‐shRNA‐ or *CDKAL1*‐shRNA‐expressing RD and RH30 cells (*n* = 4, error bars indicate mean ± SD). D) Flow cytometry analysis showing the effect of *CDKAL1* knockdown on CD133 expression in RD cells and RH30 cells. E) Immunofluorescent imaging of myogenic differentiation of RMS cells after *CDKAL1* knockdown, as indicated by positive staining for myosin heavy chain (MyHC). F) One million Control‐shRNA‐ or *CDKAL1*‐shRNA‐expressing RMS‐YM cells were subcutaneously injected at the backs of BALB/c‐nu/nu mice, and tumor growth was measured. Ten injection sites were measured for each group (error bars indicate mean ± SD). G) Immunofluorescent analysis using anti‐CD133 and anti‐MyHC antibodies in the RMS‐YM xenografts obtained in Figure 2E. All scale bars, 50 µm. H) Effect of the forced expression of CDKAL1 on the sphere‐forming capacity of RD and ICH‐ERMS‐1 cells (*n =* 4, error bars indicate mean ± SD).

Given the observed association between CDKAL1 overexpression and CSC‐related signatures, we next asked whether CDKAL1 is essential for the maintenance of RMS CSCs. We knocked down *CDKAL1* in RMS cell lines and observed that the self‐renewal and colony formation capacity and the cellular proliferation were attenuated in the *CDKAL1* knockdown lines (**Figure** **2**A–C). We also confirmed that the *CDKAL1* deficit induced differentiation, as shown by the loss of CD133 and the gain of myosin heavy chain (MyHC) both in vitro and in vivo (Figure 2D,E,G), and attenuated the tumor‐propagating potential of RMS‐YM cells in immunocompromised mice (Figure 2F). Moreover, we observed an increased self‐renewal capacity of RD and ICH‐ERMS‐1 cells upon exogenous *CDKAL1* expression (Figure 2H). These data clearly show that CDKAL1 plays a pivotal role in maintaining CSC‐related properties in RMS.

To test whether CDKAL1 impacts clinical outcomes in other cancers, we analyzed public gene expression datasets obtained from The Cancer Genome Atlas (TCGA). We found that the prognosis of CDKAL1^high^ patients, like in RMS, was significantly worse than that of the CDKAL1^low^ patients in melanoma, liver cancer, prostate cancer, stomach cancer, and glioma (Figures S1–S5, Supporting Information). To examine whether CDKAL1 was crucial for maintaining CSC properties of these cancers, we prepared two or three cell lines per type. We observed that the self‐renewal and/or colony‐forming capacity was attenuated after *CDKAL1* knockdown (Figures S1–S5, Supporting Information). We further validated that *CDKAL1* deficiency resulted in the loss of the undifferentiated state, as shown by the loss of canonical CSC‐markers in each cancer type in vitro (Figures S1–S5, Supporting Information). These findings indicate that CDKAL1 is also crucial to sustain the CSC‐related traits various cancers.

### 2.3 CDKAL1‐Dependent Mechanism Underlying the Maintenance of CSC Properties Is Dispensable for Normal Cells, but Indispensable for Transformed Cells

**Figure 3:**
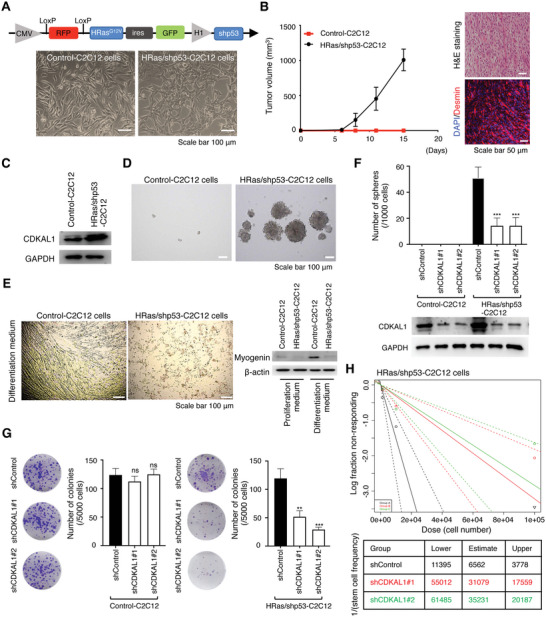
Myoblastic C2C12 cells acquire the CSC‐related properties that require CDKAL1 upon malignant transformation. A) Ectopic expression of *HRas* ^G12V^ and *tp53*‐shRNA transforms C2C12 cells. B) Tumor‐propagating capacity of transformed C2C12 cells. Note that the tumor consists of a mixed population of highly atypical undifferentiated and spindle‐shaped cells with eosinophilic cytoplasm that were positive for desmin immunostaining. These findings are consistent with the pathology of embryonal RMS. Scale bars, 50 µm. C,D) Transformed C2C12 cells showed elevated expression levels of CDKAL1 and acquired the ability of anchorage‐independent growth. Scale bars, 100 µm. E) HRas/sh*p53*‐C2C12 cells are insensitive to the differentiation cue, while Control‐C2C12 cells form myotubes and express the myogenin protein, a master regulator of myodifferentiation. Scale bar, 100 µm. F,G) The self‐renewal (F, *n =* 4, error bars indicate mean ± SD) and colony‐forming (G, *n =* 3, error bars indicate mean ± SD) capacity of Control‐shRNA‐ or *Cdkal1*‐shRNA‐expressing Control‐C2C12 or HRas/sh*p53*‐C2C12 cells. H) Limiting dilution assay displaying the tumor‐propagating capacity of HRas/sh*p53*‐C2C12 cells with Control‐shRNA or *Cdkal1*‐shRNA.

Thus far, we showed that CDKAL1 promoted CSC‐related traits maintenance in various cancers. A previous study has found that *Cdkal1* knockout mice develop and grow normally except for an elevated susceptibility to type II diabetes,^[^ ^29^ ^]^ suggesting that CDKAL1 is not essential for maintaining somatic cells in normal tissues. We next asked whether the mechanism of CSC maintenance by CDKAL1 is limited to cancer cells. To address this, we adopted a mouse myoblast cell line C2C12 because this cell line can be used to compare the normal myoblast state and RMS‐like state upon oncogenic transformation.^[^ ^33^, ^34^ ^]^ We prepared Control‐C2C12 and HRas/shp53‐C2C12 (transformed with the combination of HRas overexpression and *tp53* knockdown) cells (**Figure** **3**A): HRas/shp53‐C2C12, but not Control‐C2C12 cells, developed a tumor that resembled ERMS in immunocompromised mouse, confirmed by histological analyses (Figure 3B). After transformation, the expression levels of *Cdkal1* were elevated, and HRas/shp53‐C2C12 cells acquired anchorage‐independent growth potential, resistance to differentiation cues, and tumor‐propagating potential (Figure 3B–E). Importantly, *Cdkal1* knockdown attenuated these properties in HRas/shp53‐C2C12, but did not affect the growth of Control‐C2C12 cells (Figure 3F–H). These data indicate that the mechanism by which CDKAL1 maintains CSCs is specific to transformed cells.

### 2.4 Identification of the CSC‐Factor SALL2 as a Target of CDKAL1‐Dependent Translation

**Figure 4:**
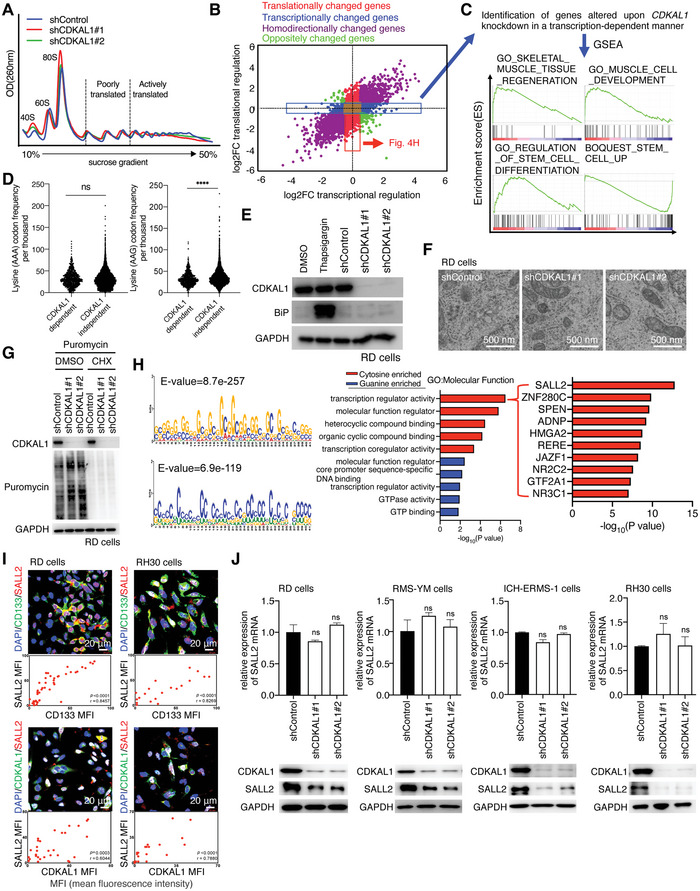
Identification of SALL2 as a CSC‐related factor whose translation is dependent on CDKAL1. A) Polysome fractionation was performed in Control‐shRNA‐ (blue line) or *CDKAL1*‐shRNA‐expressing (red or green) RD cells. Note that there was no significant change in fractional pattern among the samples. B) Identification of the genes whose transcription (blue dots) or translation (red dots) was altered by *CDKAL1* knockdown. The genes whose expression levels in total RNA were altered upon CDKAL1 knockdown are considered transcriptionally changed genes (X‐axis) and those in polysome RNA are considered translationally changed genes (Y‐axis). Homodirectionally changed genes and oppositely changed genes are shown as purple dots and green dots, respectively. C) GSEA was performed with the genes altered after CDKAL1 knockdown in a transcription‐dependent manner in RD cells (upper, blue dots). D) Informatics analysis of lysine codon usage in CDS between the CDKAL1‐dependent genes and all other genes (error bars indicate mean ± SD). E) Western blotting analysis of the ER‐stress marker BiP in Control‐shRNA‐ or *CDKAL1*‐shRNA‐expressing RD cells. Thapsigargin‐treated cells were used as a positive control of ER‐stress. F) Representative electron microscopy images of Control‐shRNA‐ or *CDKAL1*‐shRNA‐expressing RD cells. Scale bars, 500 nm. G) Puromycin incorporation assay was done to monitor the global protein synthesis in Control‐shRNA‐ or *CDKAL1*‐shRNA‐expressing RD cells. H) Identification of two *de novo* motifs, guanine‐enriched sequences (GESs) and cytosine‐enriched sequences (CESs), in the 5’UTR of the CDKAL1‐dependent mRNAs. GO term analysis showing that compared to GESs, CESs correlated highly with the transcription regulator activity. SALL2 was found at the top of gene list of “transcription regulator activity”, whose 5’UTR was correlated with the CESs. I) Immunofluorescent analysis using anti‐CD133, anti‐SALL2, and anti‐CDKAL1 in RD and RH30 cells. Scale bars, 20 µm. J) Effect of *CDKAL1* knockdown on SALL2 expression levels in RMS cell lines (*n =* 2, error bars indicate mean ± SD).

CDKAL1 catalyzes the methylthiolation of t^6^A to generate ms^2^t^6^A specifically at position 37 (A^37^), 3’ adjacent to the anticodon of mammalian cytosolic tRNA^Lys^ (UUU).^[^ ^29^, ^35^ ^]^ This modification is required to prevent misreading of the corresponding codons, which results in aberrant protein synthesis that can cause endoplasmic reticulum (ER)‐stress under the elevated translation rate.^[^ ^29^ ^]^ Based on this canonical function, we expected CDKAL1 to promote the synthesis of CSC‐related factors by increasing the elongation rate through the tRNA^Lys^ (UUU) modification. Therefore, to identify the group of genes whose translation is controlled by CDKAL1, we performed RNA sequence analysis of total RNA and RNA from the actively translated polysome fractions in RD cells expressing Control‐shRNA or *CDKAL1*‐shRNA (**Figure** **4**A), and identified genes whose expression was determined by the transcription process (Figure 4B, blue dots: by comparing the total RNA sequence results) and the translation process (Figure 4B, red dots: by comparing the polysome RNA sequence) after *CDKAL1* knockdown. It is noteworthy that the expression levels of genes associated with skeletal muscle development and stem cell differentiation were increased (Figure 4C). In contrast, those associated with stem cell enrichment were attenuated in a transcription‐dependent manner after CDKAL1 knockdown (Figure 4C), confirming that CDKAL1 deficiency impaired the maintenance of the undifferentiated state of RMS cells (Figure 2C,D). Surprisingly, the lysine content of the mRNAs whose translation was CDKAL1‐dependent was not significantly different (AAA codon) from or less (AAG codon) than that of CDKAL1‐independent mRNAs (Figure 4D), suggesting that the mechanism by which CDKAL1 maintains cancer cell stemness is independent of the known function of CDKAL1. Moreover, unlike a previous report, CDKAL1 deficiency did not induce ER‐stress in RD cells, as validated by the lack of induction of the ER‐stress marker BiP and the lack of the enlargement of ER lumen detected by electron microscopy (Figure 4E,F). Interestingly, by a puromycin incorporation assay that measures the intracellular global protein synthesis, CDKAL1 deficiency did not attenuate global protein synthesis in RD cells (Figure 4G). These results suggest that CDKAL1 maintenance of the CSC‐related traits is independent of the canonical role of a tRNA‐modifying enzyme that controls the elongation phase.

**Figure 5:**
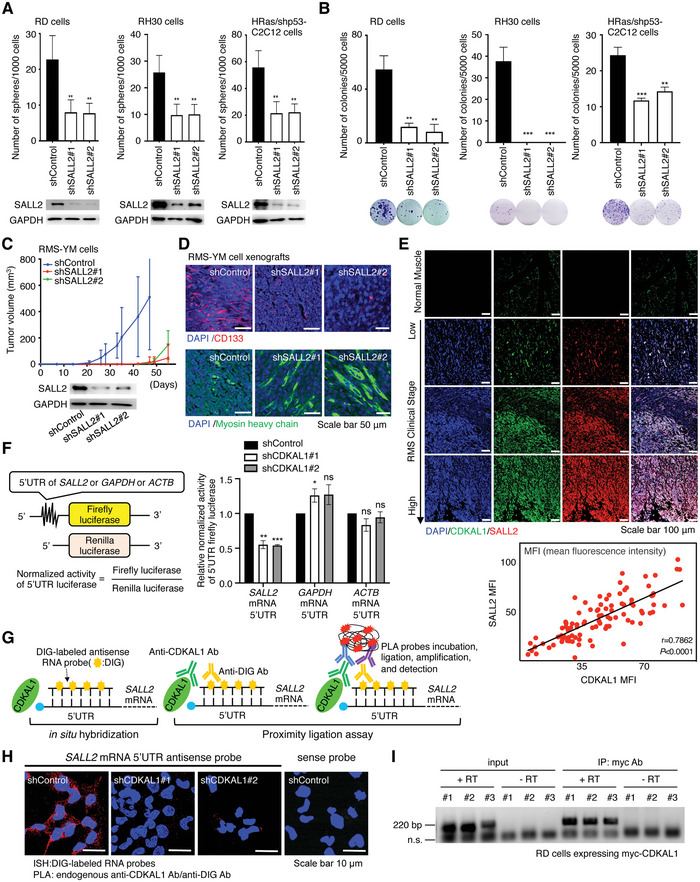
SALL2 is an essential factor for maintaining CSCs in RMS and is controlled by CDKAL1 at the translation initiation phase. A,B) The self‐renewal capacity (A, *n = 4*, error bars indicate mean ± SD) and colony‐forming potential (B, *n =* 3, error bars indicate mean ± SD) of Control‐shRNA‐ or *SALL2*‐shRNA‐expressing RMS cells were measured (*n* = 4, error bars indicate mean ± SD). Western blotting results are shown to confirm *SALL2* knockdown (A). C) One million Control‐shRNA‐ or *SALL2*‐shRNA‐expressing RMS‐YM cells were injected subcutaneously at the backs of BALB/c‐nu/nu mice, and tumor growth was measured. Ten injection sites were measured for each group (error bars indicate mean ± SD). D) Immunofluorescent analysis using anti‐CD133 and anti‐MyHC antibodies in the RMS‐YM xenografts obtained in Figure 5C. All scale bars, 50 µm. E) Immunofluorescent analysis of normal and pathological specimens using anti‐CDKAL1 and anti‐SALL2 antibodies. Note that CDKAL1^high^ tumor cells exhibit SALL2^high^ properties in RMS. Scale bars, 100 µm. F) Luciferase reporter analysis with 5’UTR‐*SALL2*, ‐*GAPDH*, and ‐*ACTB* in Control‐shRNA‐ or *SALL2*‐shRNA‐expressing RD cells (*n =* 3, error bars indicate mean ± SD). G) A cartoon representing the procedure. H) In situ proximity ligation assay detecting a signal obtained by the reactions from endogenous CDKAL1 and the DIG‐labeled sense or antisense RNA probes of the 5’UTR region of *SALL2* mRNA in Control‐shRNA‐ or *SALL2*‐shRNA‐expressing RD cells. Scale bars, 10 µm. I) RNA immunoprecipitation and reverse transcription PCR assay using RD cells expressing myc‐tagged CDKAL1. Primer pairs for 5’UTR of *SALL2* mRNA were used to detect the interaction between CDKAL1 and *SALL2* mRNA. Reverse transcriptase (‐) samples serve as negative controls. Three samples (#1, #2, and #3) were analyzed.

To determine how CDKAL1 regulated the translation of these mRNAs, we then analyzed the 5’ untranslated region (5’UTR) of the mRNAs whose translation was CDKAL1‐dependent. MEME (Multiple Em for Motif Elicitation) analysis showed that guanine‐enriched sequences (GESs) and cytosine‐enriched sequences (CESs) were enriched in the 5″UTR of CDKAL1‐dependent mRNAs (Figure 4H). Gene Ontology (GO) analysis indicated that the mRNAs with CESs were more highly correlated with the GO term for “transcription regulator activity” than those with GESs. Since transcription factors can be master regulators of stem cell activity in various cancers,^[^ ^3^, ^4^, ^5^ ^]^ we searched for CDKAL1‐dependent mRNAs containing CESs assigned to the GO term “transcription regulator activity.” We found *SALL2* mRNA at the top of the gene list whose 5’UTR best correlated with the CES we identified (Figure 4H). SALL2 is a core transcription factor network component that maintains cancer stemness in glioblastoma,^[^ ^4^ ^]^ but its role in other cancers, including RMS, is unclear. We confirmed the overlapped immunofluorescent signals of CDKAL1 and SALL2 in RMS cells (Figure 4I), as well as melanoma (Figure S6A, Supporting Information), liver cancer (Figure S7A, Supporting Information), prostate cancer (Figure S8A, Supporting Information), and stomach cancer (Figure S9A, Supporting Information). SALL2 protein levels were reduced in a translation‐dependent manner after *CDKAL1* knockdown in RMS cell lines (Figure 4J). We found that *SALL2* knockdown significantly reduced the self‐renewal and colony‐forming capacity and tumor‐propagating potential (**Figure** **5**A–C). In RMS, we also confirmed that *SALL2* deficiency induces differentiation, as indicated by the loss of CD133 and the gain of MyHC signals (Figure 5D). These findings were also observed in other cancer cell lines (Figures S6–S10, Supporting Information). We also immunostained RMS patient specimens and found that *SALL2* expression levels correlated with *CDKAL1* (Figure 5E). To test whether CDKAL1 regulates *SALL2* translation, we fused a luciferase reporter with the *SALL2* 5″UTR and found that the activity of the reporter was reduced after *CDKAL1* knockdown in RD cells, whereas that with *GAPDH* or *ACTB* 5″UTR was not (Figure 5F). Furthermore, an in situ proximity ligation assay (PLA) using anti‐CDKAL1 and anti‐digoxigenin (DIG) antibodies and in situ hybridization of DIG‐labeled sense/antisense *SALL2* 5″UTR sequences showed that the CDKAL1 protein and RNA antisense probe signals were reduced after *CDKAL1* knockdown (Figure 5G,H). We also performed RNA immunoprecipitation and reverse transcription PCR assay using RD cells expressing myc‐tagged CDKAL1 and confirmed the interaction between CDKAL1 protein and *SALL2* 5'UTR sequence (Figure 5I). These data suggest that the expression level of *SALL2*, an essential factor for maintaining CSCs in RMS and other cancers, is controlled by CDKAL1 at the translation initiation phase.

### 2.5 CDKAL1 Promotes Assembly of the eIF4F Translation Initiation Complex

**Figure 6:**
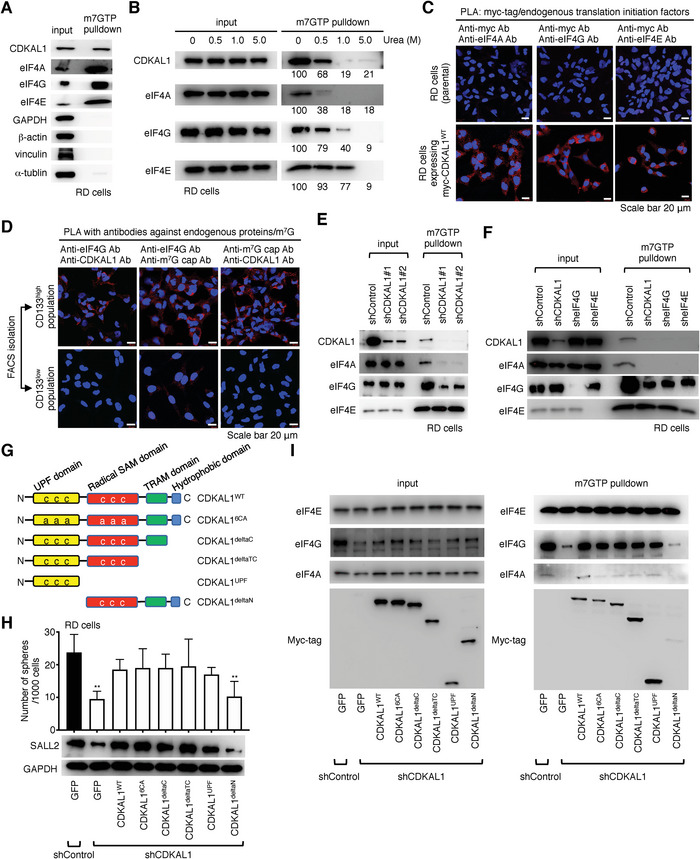
CDKAL1 promotes the assembly of the eIF4F translation initiation complex. A) Western blotting analysis of m^7^GTP precipitate from RD cell lysate. GAPDH, *β*‐actin, vinculin, and *α*‐tubulin serve as negative controls. B) Western blotting analysis of m^7^GTP precipitate from RD cell lysate with urea‐based denaturation. Numbers indicate the densitometry of signals (percent of control, Urea 0 m). C) An in situ proximity ligation assay using anti‐myc tag antibody and antibodies against endogenous eIF4A, eIF4G, and eIF4E in parental RD cells and RD cells expressing myc‐CDKAL1^WT^. Scale bars, 20 µm. D) An in situ proximity ligation assay using antibodies against endogenous proteins and m^7^G cap. Note that CD133^high^ RD cells showed elevated expression levels of CDKAL1 compared to CD133^low^ RD cells (Figure 1E). E) Western blotting analysis of m^7^GTP precipitate from Control‐shRNA‐ or *CDKAL1*‐shRNA‐expressing RD cell lysate. F) Western blotting analysis of m^7^GTP precipitate from Control‐shRNA‐, *CDKAL1*‐shRNA‐, *EIF4G*‐shRNA‐, *EIF4E*‐expressing RD cell lysate. G) A cartoon representing the mutants used in this study. H) Results of the rescue experiment with several truncated mutants of CDKAL1 to maintain the self‐renewal capacity and SALL2 protein levels of RD cells (*n =* 4, error bars indicate mean ± SD). I) The m^7^GTP pull‐down experiments with *CDKAL1*‐shRNA‐expressing RD cells displaying that the NH_2_‐terminus of CDKAL1 is essential for sustaining the eIF4F complex formation.

Next, we sought to determine the mechanism by which CDKAL1 regulates *SALL2* translation. The translation of mRNAs with highly complex structured 5’UTR such as CG‐rich sequences requires the helicase activity of the translation initiation factor complex (eIF4E/eIF4A/eIF4G, hereafter eIF4F).^[^ ^7^, ^8^, ^36^, ^37^ ^]^ We, therefore, asked whether CDKAL1 promoted the translation of *SALL2* mRNA by regulating the function of the eIF4F complex. To test this, we performed m^7^GTP pull‐down experiments with RD cell extracts and found that CDKAL1 coprecipitated with eIF4A and eIF4G, the legitimate partners of eIF4E (**Figure** **6**A). m^7^GTP pull‐down experiment with urea‐based denaturation demonstrated that CDKAL1 was found in the precipitate more stable than eIF4A, suggesting that CDKAL1 was bound to eIF4E or eIF4G (Figure 6B). For further validation, we performed PLA in parental RD cells or myc‐tagged CDKAL1‐expressing RD cells and confirmed the in situ interaction of myc‐tagged CDKAL1 and endogenous eIF4E, eIF4A, and eIF4G (Figure 6C). Importantly, PLA signals from the antibody pairs of endogenous CDKAL1 and eIF4G, eIF4G and m^7^G cap, and endogenous CDKAL1 and m^7^G cap, were detected at much higher levels in CD133^high^ RD cells than CD133^low^ RD cells, probably because of the elevated expression levels of CDKAL1 in CD133^high^ RD cells (Figure 6D). Surprisingly, *CDKAL1* knockdown markedly attenuated the coprecipitation of eIF4A and eIF4G by m^7^GTP beads (Figure 6E), and this was also confirmed in the glioblastoma initiating cells MGG8, and MGG18 (Figure S10D, Supporting Information). *EIF4G* knockdown attenuated the pull‐down of CDKAL1 by m^7^GTP beads (Figure 6F). These data indicate that CDKAL1 is essential for promoting eIF4F complex formation, probably in cooperation with eIF4G, and that CDKAL1 incorporation in the eIF4F complex is abundant in the CSC population.

To identify the region of CDKAL1 that is essential for the eIF4F complex formation, we generated the truncated mutants of CDKAL1 (Figure 6G) and tested whether they could rescue the reduced capacity for self‐renewal and decreased SALL2 protein levels of *CDKAL1*‐shRNA‐expressing RD cells. We found that CDKAL1^6CA^, which lacks methylthiolation activity, could restore these phenotypes to levels comparable to those seen with wild‐type CDKAL1, suggesting that eIF4F complex formation was independent of the methylthiolation enzymatic activity of CDKAL1 (Figure 6H). CDKAL1^deltaC^, CDKAL1^deltaTC^, and CDKAL1^UPF^, which lack the hydrophobic region of COOH‐terminus, the TRAM domain, and the radical SAM domain, respectively, but not CDKAL1^deltaN^, which lacks the NH_2_‐terminus, also rescued these phenotypes and eIF4F complex formation (Figure 6H,I). These data indicate that the NH_2_‐terminus of CDKAL1 is an important region for the eIF4F complex formation.

**Figure 7:**
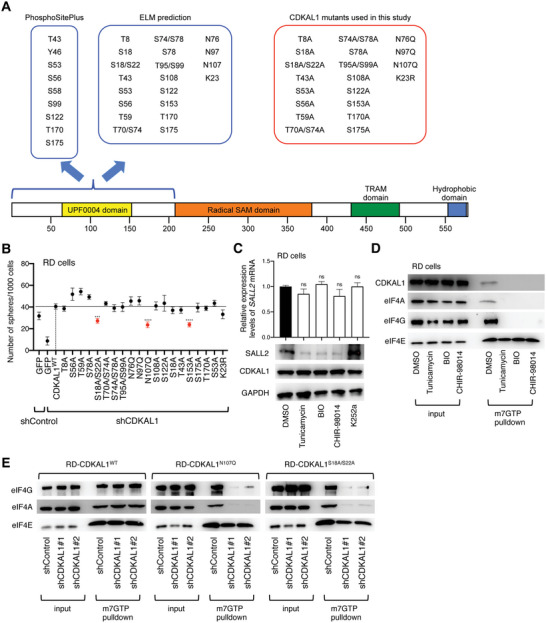
Identification of sites in the NH_2_‐terminus domain of CDKAL1 essential for forming the eIF4F complex. A) A graphical summary of the candidate amino acid mutations in the NH_2_‐terminus of CDKAL1. B) Sphere‐formation assay screening with a series of full‐length CDKAL1 with mutants for possibly modified amino acid(s) (*n* = 4, error bars indicate mean ± SD). C) Effect of inhibitors of *N*‐glycosylation (tunicamycin), GSK3 kinase (BIO and CHIR‐98014), and K252a (phosphorylase kinase inhibitor) on SALL2 protein expression levels in RD cells. Quantitative PCR result shows no reduction in *SALL2* mRNA expression after treatment with these inhibitors (*n =* 2, error bars indicate mean ± SD). D) Effect of tunicamycin, BIO, and CHIR‐98014 on the CDKAL1 incorporation in the eIF4F complex. E) The m^7^GTP pull‐down experiments with *CDKAL1*‐shRNA‐expressing RD cells displaying that the N107 and S18/S22 amino acid residues of CDKAL1 are essential for sustaining the eIF4F complex formation.

Because the NH_2_‐terminus of CDKAL1 contains several predicted sites of post‐translational modifications that could alter the protein–protein interactions, as demonstrated by either ELM motif analysis or PhosphoSitePlus data collection (**Figure** **7**A), we hypothesized that modifications in the NH_2_‐terminus of CDKAL1 could be essential for the eIF4F complex formation. To test this, we generated a series of full‐length CDKAL1 mutants with candidate mutations in the NH_2_‐terminus domain (Figure 7A). We examined whether these mutants could rescue the reduced self‐renewal capacity of *CDKAL1*‐shRNA‐expressing RD cells and found that CDKAL1^S18A/S22A^, CDKAL1^N107Q^, and CDKAL1^S153A^ mutants could not fully rescue the phenotype (Figure 7B); because these mutants harbored the mutations in the amino acid(s) of the potential modification site of phosphorylation by GSK3, *N*‐glycosylation, and phosphorylation by phosphorylase kinase, respectively, we then tested the effects of the inhibitors of these enzymes. Treatment with tunicamycin (*N*‐glycosylation inhibitor), BIO (GSK3 inhibitor), and CHIR‐98014 (GSK3 inhibitor) significantly reduced SALL2 levels in RD cells, but K252a (phosphorylase kinase inhibitor) did not (Figure 7C). We further found that pretreatment of RD cells with tunicamycin, BIO, or CHIR‐98014 abolished the incorporation of CDKAL1 into the eIF4F complex, resulting in dissociation of the complex (Figure 7D). We observed that both CDKAL1^N107Q^ and CDKAL1^S18A/S22A^ mutants could not rescue the dissociation of the eIF4F complex induced by CDKAL1 knockdown (Figure 7E). These data indicate that the post‐translational modification of the NH_2_‐terminus domain of CDKAL1 is important in promoting eIF4F complex formation.

## 3 Discussion

CDKAL1 has been spotlighted in the association of the SNPs with an increased risk of type II diabetes.^[^ ^26^, ^27^, ^28^ ^]^ Because of the canonical role of CDKAL1 that converts t^6^A to ms^2^t^6^A at the 37^th^ adenosine in tRNA^Lys^ (UUU), loss of CDKAL1 function results in decreased ms^2^t^6^A modification in tRNA^Lys^ (UUU), leading to a misreading of the lysine codon of the proinsulin gene and decreased insulin secretion.^[^ ^29^ ^]^ Although many reports have been published on the association of CDKAL1 with diabetes,^[^ ^38^, ^39^, ^40^ ^]^ no biological evidence has linked CDKAL1 to cancer progression. Here, we unveiled the pivotal role of CDKAL1 in cancer biology, which was independent of the canonical role of CDKAL1 as an RNA‐modifying methyltransferase. We found that the expression levels of CDKAL1 were increased in the population of CSCs and demonstrated that CDKAL1 was required for the maintenance of CSCs‐related traits in RMS as well as the other common cancers, such as melanoma, liver cancer, prostate cancer, stomach cancer, and glioma (Figures 1 and 2 and Figures S1–S5, Supporting Information). Furthermore, CDKAL1 expression levels in these cancers were associated with a worse prognosis. Importantly, by comparing normal myoblast C2C12 cells and oncogene‐transformed C2C12 cells, we showed that the mechanism by which CDKAL1 maintained CSCs was dispensable for normal somatic cells but essential for the malignantly transformed cells (Figure 3). This is consistent with our previous observation that the *Cdkal1*‐knockout mouse showed no developmental failure.^[^ ^29^ ^]^

As the molecular mechanism by which CDKAL1 maintained the CSCs, we discovered a non‐canonical role of CDKAL1 in the translation process, especially at the initiation phase. CDKAL1 deficit attenuated the translation of selective mRNAs whose 5’UTR contained CES and/or GES and could thus be highly structured (Figure 4), without inducing a global loss of protein synthesis (Figure 4G). Mechanistically, CDKAL1 promotes the assembly of the eIF4F translation initiation complex (Figure 6). This complex formation does not require the enzyme activity of CDKAL1 but requires only the NH_2_‐terminus domain of CDKAL1 (Figure 6G–I). Biochemistry experiments further confirmed the post‐translational modification sites in CDKAL1 that were essential for CSC maintenance (Figure 7). Notably, both GSK3 phosphorylation and N‐glycosylation enrichment are involved in CSC behavior.^[^ ^41^, ^42^ ^]^ Our findings suggest that CDKAL1 is incorporated into the eIF4F translation initiation complex by the protein‐protein interaction between CDKAL1 and the complex, probably through the modified amino acid residues in CDKAL1, thus promoting the translation of selective mRNAs.

**Figure 8:**
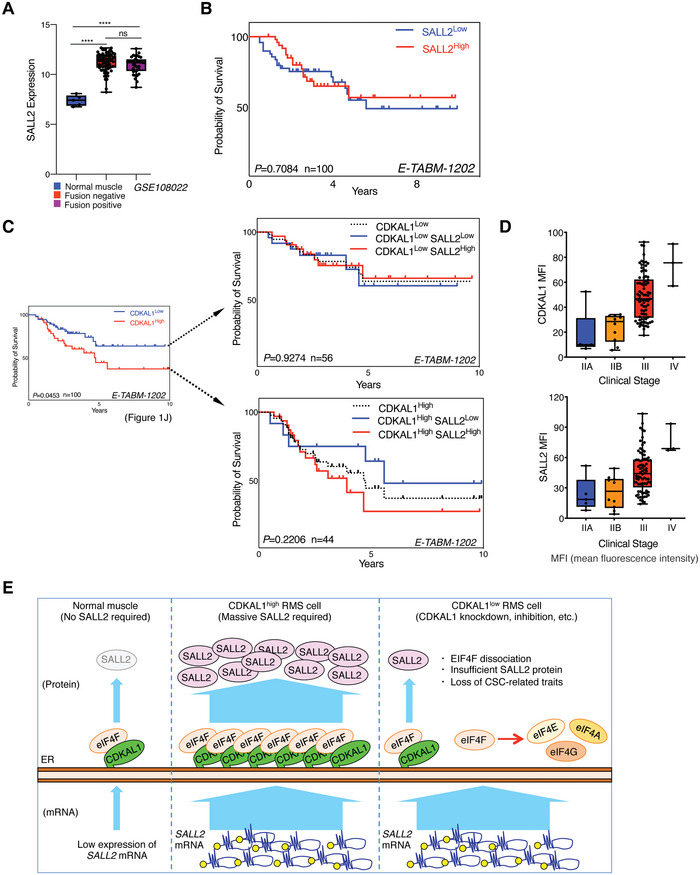
CDKAL1 overexpression is associated with a worse prognosis in SALL2^high^ RMS patients. A) *SALL2* mRNA expression levels in normal skeletal muscle and fusion‐negative and fusion‐positive RMS in a gene set of GSE108022 are shown. B) Kaplan–Meier curve displaying the prognosis in RMS patients with SALL2^high^ and SALL2^low^ signature from the E‐TABM‐1202 dataset. C) Kaplan–Meier curve showing that elevated CDKAL1 expression levels worsen the prognosis of the SALL2^high^ population of RMS patients. D) The expression levels of CDKAL1 and SALL2 in RMS patients at various clinical stages. E) Graphical summary of the roles of CDKAL1 in normal tissue and CSCs.

Among the target genes of a CDKAL1‐dependent translation mechanism, we demonstrated that SALL2 was crucial for maintaining CSCs of all cancers mentioned above (Figure 5 and Figures S6–S10, Supporting Information). *SALL2* mRNA expression was significantly higher in RMS specimens than in normal skeletal muscle (**Figure** **8**A), and this was accompanied by elevated levels of *CDKAL1* mRNA expression (Figure 1H). Although the expression levels of *SALL2* mRNA do not predict prognosis (Figure 8B), intriguingly, the CDKAL1^high^ signature tends to be associated with a worse prognosis in the SALL2^high^ population (Figure 8C). The expression levels of both *CDKAL1* and *SALL2* were positively correlated with each other (Figure 5E) and increased as the clinical stage progressed (Figure 8D). These results suggest that CDKAL1 determines the translation capacity of *SALL2* mRNA, which is essential for the maintenance of CSC‐related properties but not essential for normal cells that do not require SALL2 protein (Figure 8E).

This study picks up SALL2 as a candidate for the CDKAL1‐dependent CSC‐related factor (Figure 4). However, among the other CDKAL1 target mRNAs whose 5’UTR contained CES and/or GES, we notice several well‐known CSC factors, including the Hippo transducer YAP1. YAP1 is essential for maintaining CSCs and a cause of poor prognosis in many cancers, including RMS.^[^ ^43^, ^44^, ^45^ ^]^ CDKAL1 deficit does not induce a global loss of protein synthesis but attenuates the translation of selective mRNAs, including the CSC factors, such as SALL2 and YAP1, and thus may synergistically inhibit the CSC properties. This suggests that CDKAL1 is a potential therapeutic target. The enzymatic inhibition of CDKAL1 might cause the development of type II diabetes, but as we show here, CDKAL1‐dependent control of the translation initiation of CSC‐related factors is independent of the enzyme activity but rather dependent on protein‐protein interaction, which several known compounds can inhibit. Our data shed light on the possibility of developing new drugs that target a CSC‐selective translation regulatory mechanism.

## 4 Experimental Section

### Cell Culture and Treatments

Human rhabdomyosarcoma cell lines RD and ICH‐ERMS‐1 were obtained from the Japanese Collection of Research Bioresources (JCRB), RMS‐YM from the RIKEN BioResource Research Center (RBRC), and RH30 from the American Type Culture Collection (ATCC). Mouse myoblast cell line C2C12 was obtained from RBRC. The human melanoma cell line A2058 was obtained from JCRB, SK‐Mel‐28 from ATCC, and HMV‐II from RBRC. Human liver cancer cell lines HuH‐7 and HepG2 were obtained from JCRB. Human prostate cancer cell lines PC3 and LNCaP were obtained from RBRC. Human stomach cancer cell lines NUGC3 and MKN45 were obtained from JCRB, and HGC27 from RBRC. Human glioblastoma cell lines MGG4, MGG8, MGG18 were kindly provided by Dr. Wakimoto (Massachusetts General Hospital). For lentivirus production, 293FT cells were purchased from Invitrogen (catalog number: R70007). RD, ICH‐ERMS‐1, RMS‐YM, RH30, C2C12, A2058, SK‐Mel‐28, HMV‐II, PC3, LNCaP, NUGC3, MKN45, HGC27, and 293FT cells were cultured at 37 °C containing 5% CO_2_ in high glucose Dulbecco's modified Eagle's medium (DMEM, Fujifilm‐Wako) supplemented with 10% fetal bovine serum (FBS, Corning) and 1% penicillin/streptomycin/L‐glutamine (Fujifilm‐Wako). HuH‐7 and HepG2 cells were grown in low glucose DMEM with 10% FBS and 1% antibiotics. MGG4, MGG8, and MGG18 cells were grown in sphere culture medium (neurobasal medium (Thermo Fisher Scientific) supplemented with 20 ng mL^‐1^ of EGF, 20 ng mL^‐1^ of bFGF, 1 × B‐27 supplement (Thermo Fisher Scientific), 1 × N‐2 supplement (Thermo Fisher Scientific), 10 µg mL^‐1^ of heparin (Sigma‐Aldrich) and 1% antibiotics). Cells were cultured in a differentiation medium (DMEM supplemented with 2% horse serum (Thermo Fisher Scientific)) to induce C2C12 cells differentiation, and the medium was changed every two days. For the sphere‐formation assay experiments, 1000 cells per well were seeded on ultra‐low attachment 24‐well plates (Corning) in 1.5 mL of sphere culture medium and cultured at 37 °C containing 5% CO_2_ for 7 d. Four technical replicates were prepared for each condition. For the single‐cell sphere‐formation assay, the single glioma cells were plated in 150 µL of sphere culture medium in 96‐well plates for each condition. After 14 d, the sphere number per 96‐well plate was assessed. For the colony formation assay experiments, cells were seeded at a density of 5000 cells per well (except ICH‐ERMS‐1 cells: 10 000 cells per well) in six‐well plates and cultured until the colonies can be clearly observed. The cells were then fixed with 4% paraformaldehyde (PFA, Fujifilm‐Wako), stained with 0.05% crystal violet (Fujifilm‐Wako), and evaluated using ImageJ software (Multi‐point tool). Three technical replicates were performed. For the drug treatment experiments, the cells were treated for 24 h with the following drug concentration below: 10 × 10^‐6^ m of thapsigargin (Sigma‐Aldrich), 1 µg mL^‐1^ of tunicamycin (Sigma‐Aldrich), 5 × 10^‐6^ m of BIO (Selleck), 5 × 10^‐6^ m of CHIR‐98014 (Selleck), and 5 × 10^‐6^ m of K252a (Abcam). DMSO was used as a negative control in each experiment.

### Plasmids, Lentivirus Preparation, and Infection

Short‐hairpin sequences were cloned into a pLKO.1 puro vector (Addgene #8453) at the restriction enzyme sites of AgeI/EcoRI to generate plasmids for shRNA‐expressing lentiviruses. For overexpression experiments, coding sequences of green fluorescent protein (GFP, used as a negative control), wild‐type CDKAL1, or mutants of CDKAL1 were cloned into a pTomo vector (Addgene #26291) at the restriction enzyme sites of XbaI/SalI. The pTomo‐HRas/shp53 used for the malignant transformation of C2C12 cells was a kind gift from Dr. Friedmann‐Morvinski (Tel Aviv University). Lentivirus particles were produced as we previously described.^[^ ^46^ ^]^ Briefly, 293FT cells with 10 µg of lentiviral backbone plasmid, 7.5 µg of psPAX2 (Addgene #12260), and 2.5 µg of pMD2.G (Addgene #12259) using TransIT‐LT1 Transfection Reagent (TaKaRa Bio) and Opti‐MEM (Thermo Fisher Scientific) were transfected. The viral particle‐containing medium was harvested after 72 h of transfection. Cells were infected with 1 mL of viral supernatant in a 60 mm cell culture dish (Thermo Fisher Scientific). For the malignant transformation, C2C12 cells were infected with pTomo‐HRas/shp53 and further infected with Cre recombinase‐expressing adenovirus (Ad‐CMV‐iCre, purchased from VECTOR BIOLABS) to activate HRas expression cassette. All recombinant DNA experiments were performed under permission from the committee of Okayama University (approval number: 17138). The following are the target sequences of shRNA used in this study:

Human CDKAL1 #1 [CTAGCTGCTTATGGCTATAAA];

Human CDKAL1 #2 [ATGCATCCGATGCAGATTTAT];

Human SALL2 #1 [GCAGTGGAACCCAAGAATAAA];

Human SALL2 #2 [CCGCTTCTGTGCCAAAGTATT];

Murine Cdkal1 #1 [GCTTGCTGCCTATGGCTATAA];

Murine Cdkal1 #2 [GCCTCCATAAGGAATAAGTTT];

Murine Sall2 #1 [GATGCAGATGACTGAACAAAT];

Murine Sall2 #2 [CGGAAGAAAGAAGAAACTATA];

Human eIF4G [GCCCTTGTAGTGACCTTAGAA];

Human eIF4E [CGGCTGATCTCCAAGTTTGAT].

### Protein Extraction and Western Blotting

Protein extract was done using lysis buffer (20 ×10^‐3^ m Tris‐HCl (pH = 7.5), 150 ×10^‐3^ m NaCl, 1 ×10^‐3^ m ethylene diamine tetraacetic acid (EDTA), 1 × ×10^‐3^ m ethylene glycol tetraacetic acid (EGTA), 0.5% Triton X‐100, cOmplete Protease Inhibitor Cocktail (Sigma‐Aldrich), and PhosSTOP phosphatase inhibitor cocktail (Sigma‐Aldrich)). After sonication, the lysate was cleared by centrifugation at 13 500 rpm and 4 °C for 15 min, and the supernatant was boiled with 4 × SDS sample buffer (240 ×10^‐3^ m Tris‐HCl (pH = 6.8), 8% sodium dodecyl sulfate (SDS), 40% glycerol, 0.1% bromophenol blue, 20% 2‐mercaptoethanol) at 95 °C for 5 min. BCA Protein Assay Kit (Thermo Fisher Scientific) was used to measure the protein concentration. Equal amounts of proteins were loaded onto acrylamide gel and transferred onto polyvinylidene fluoride (PVDF) membranes (Immobilon‐P, 0.45 µm, Millipore). The membranes were blocked with 0.5% skim milk (Nacalai Tesque) in Tris‐buffered saline with tween20 (TBST: 137 ×10^‐3^ m NaCl, 2.68 ×10^‐3^ m KCl, 25 ×10^‐3^ m Tris (pH = 7.4), and 0.1% Tween20) at room temperature for 1 h and were incubated with primary antibodies for overnight at 4 °C. After a brief wash with TBST three times, the membranes were incubated with HRP‐conjugated secondary antibodies for 1 h at room temperature. The signals were developed with Clarity Western enhanced chemiluminescence (ECL) Substrate (Bio‐Rad Laboratories). The following are the antibodies used in this study, listed as [Protein/Source/Identifier]: [GAPDH/Proteintech/ 60004‐1‐Ig]; [CD133/Proteintech/18470‐1‐AP]; [CD133/Proteintech/66666‐1‐Ig]; [CD133/BioLegend/372808]; [CDKAL1/Proteintech/22988‐1‐AP]; [CDKAL1/Santa Cruz Biotechnology/sc‐393447]; [Myosin Heavy Chain/R&D Systems/MAB4470]; [Myc tag/Medical&Biological Laboratories/M192‐3]; [ALDH1/Novus Biologicals/NBP1‐89152]; [CD44/BioLegend/103001]; [SOX2/Santa Cruz Biotechnology/sc‐17320]; [POU3F2/Cell Signaling Technology/12137S]; [SALL2/Bethyl Laboratories/A303‐208A]; [Desmin/Novus Biologicals/NBP1‐45143]; [Myogenin/Santa Cruz Biotechnology/sc‐12732]; [*β*‐Actin/Cell Signaling Technology/3700S]; [eIF4A1/Cell Signaling Technology/2490S]; [eIF4G1/Proteintech /15704‐1‐AP]; [eIF4E/Cell Signaling Technology/2067S]; [Digoxigenin/Roche/11333062 910]; [7‐methylguanosine (m7G)‐Cap/Medical&Biological Laboratories/RN016M]; [OLIG2/Abcam/ab109186]; [BiP/Cell Signaling Technology/3177S]; [Puromycin/Sigma‐Aldrich/618582]; [Donkey anti‐Rabbit IgG, Alexa Fluor Plus 594/Thermo Fisher Scientific/A32754]; [Donkey anti‐Mouse IgG, Alexa Fluor 488/Thermo Fisher Scientific/A21202]; [Donkey anti‐Rabbit IgG, Alexa Fluor Plus 488/Thermo Fisher Scientific/A21206]; [Donkey anti‐Mouse IgG, Alexa Fluor 594/Thermo Fisher Scientific/A21203]; [Donkey anti‐Rat IgG, Alexa Fluor 647/Abcam/ab150155]; [Donkey anti‐Rat IgG, Alexa Fluor 594/Thermo Fisher Scientific/A21209]; [Anti‐Rabbit IgG, HRP‐linked/Cell Signaling Technology/7074P2]; [Anti‐Mouse IgG, HRP‐linked/Sigma‐Aldrich/A9044]; [Anti‐Goat IgG, HRP‐linked/Sigma‐Aldrich/A4174]; [Anti‐Rat IgG, HRP‐linked/Sigma‐Aldrich/A5795].

### Surface Sensing of Translation (SUnSET) Experiment

A puromycin incorporation assay, also known as the surface sensing of translation (SUnSET) assay, was performed as described with a modification.^[^ ^47^ ^]^ Briefly, after pretreatment with DMSO or 100 µg mL^‐1^ of cycloheximide (CHX) (Sigma‐Aldrich) for 30 min, the cells were cultured in a growth medium containing 10 µg/mL of puromycin (Sigma‐Aldrich) with or without 100 µg mL^‐1^ of CHX for 30 min. The medium was then replaced with a puromycin‐free growth medium with or without 100 µg mL^‐1^ of CHX and cultured for 1 h. The treated cells were subjected to western blotting analysis using an anti‐puromycin antibody.

### Quantitative Polymerase Chain Reaction (PCR) for Gene Expression Analysis

The TRIzol reagent (Invitrogen) extracted total RNAs from cells by following the manufacturer's instructions. The RNAs were treated with DNase I (TaKaRa Bio) and were subjected to reverse transcription reaction using PrimeScript RT Master Mix (TaKaRa Bio). Quantitative real‐time PCR was done using SYBR Green Master (New England Biolabs) and analyzed with Rotor‐Gene Q 2plex HRM system (Qiagen). The expression level of each gene was normalized with that of ribosomal RNA 18S and analyzed with two technical and biological replicates. The following are the sequences of oligonucleotides used in this study, listed as [Gene name; forward; reverse]: [CDKAL1; TTCTTGACCGACTGAGACCCA; TCATGTTCTCCAACGCCTCTT]; [KIAA1456; ACAATGTCTGTCGAAGATCCAC; CCCTTCTCGAAACACATGGTAG]; [DUS1L; GGGAGCATATCAAGGCTGTG; GCACGATGTCCAGATACTCCT]; [TRUB2; AGCTACAGATGACTTCCGTGA; GCCTTGGATAACGGCCAGA]; [TRMU; CAGATGCCATTGCCACAGGT; TTAACGTGCTTCTGCTCAAAGA]; [TRMT11; AGACCCTCGTTTTAGGTGGAA; TGTGAATACTGGTCCCGATTCTC]; [TRMT10C; ACATAGCAATGGGCTGGAAG; TCTCTGTGCAAAGCACCATC]; [TRMT10A; GGGAGACCAGCACTGGATTA; ACCTTCTTGCCACCAACATC]; [TRMT1; ACCGAGTTTGCTCGCATTCA; CGACCACTTTTTGCGTGTCC]; [PUS10; CCCAAGAAAATTCGACTGCAAG; CTCAACCTTTTGGCACACCTT]; [TRMT2A; ACCCCAGCAGACTGAGTATC; GCCCACGGTGTTATCCTCC]; [TRMT2B; GAGCCCTGCCTTGTATTTCAT; ACGGGTCATGGTACTTTCCTG]; [LAGE3; GGCCGCACATATTCACCCTC; AGTTGATGACGGAAATTCGGAG]; [OSGEP; GTATAGGCCACATTGAGATGGG; ACCTGCGTATTTCCTCCACTC]; [TP53RK; TCCAGAGGATAAGGGAGTAGACC; GAGGAGGTGGAGTAGCTCTTC]; [TRIT1; GTGATTGACCGAAAAGTGGAGC; CCGTATGTTGACGATGGAGAAAT]; [YRDC; GGGACAAATTGGGGATGGC; GCACAGCCTGGACGAATGA]; [TRMT5; CCAGGCATAAGACGTGTGATT; CCTGCTAAACCCTGAAGTTACAT]; [WDR4; CTTGGTGGCCGACAAGTCT; GAAGCGGTCATCAGGACTCAC]; [TRDMT1; TGCCAAGACGATTGAAGGCAT; GCAGGGAGGGCTCATTAAAAT]; [TRMT12; AAATCTGGGACCGGAACTCTG; ATACCCGCCCTCGTTTTGC]; [CDKAL1; GGGACTGAGTATCATTGGGGT; CCAAGCCGCCTTCCATTATC]; [ELP4; ATGGGCATACTTTGTTGGTTGC; ACTGGTAACGCCAAGCTATTTT]; [ELP3; ACGAGGCAGTCAAGTATTCTGA; GCAGTAATCTGGTCTGGTTTCA]; [IKAP; TTTGCCCTGGGATGACCATAG; TCCACACTCTGACCTTCCGAG]; [ALKBH8; TTAATGCCACCTAACAAGCCG; ATTGAGGGTAACATAGGCTCTCT]; [ADAT3; GCGGCACCTACGACTTCAG; CCTCGTCTGCGTCCAGTTTAC]; [NSUN2; GAACTTGCCTGGCACACAAAT; TGCTAACAGCTTCTTGACGACTA]; [FTSJ1; TCCCACCCGCATCATTGTG; AGTGTACTTGTACTCTGAGCCG]; [SALL2; CCCCTGATCTTGGAAGAGCTA; CACCGTCTGGCCTAAGGAG]; [18S rRNA; GTAACCCGTTGAACCCCATT; CCATCCAATCGGTAGTAGCG].

### Fractionation and Isolation of Polysome‐Containing RNAs

The polysome fractionation was done as previously reported.^[^ ^48^ ^]^ Briefly, the cells were treated with 100 µg mL^‐1^ of CHX in a culture medium at 37 °C for 20 min. After washing with phosphate‐buffered saline (PBS) containing 100 µg mL^‐1^ of CHX, the cells were lysed in polysome lysis buffer (100 ×10^‐3^ m KCl, 5 ×10^‐3^ m MgCl_2_, 0.5% NP‐40, 2 ×10^‐3^ m DTT, 100 µg mL^‐1^ cycloheximide, 20 × 10^‐3^ m HEPES (pH = 7.4), and cOmplete Protease Inhibitor Cocktail) on ice for 30 min. The lysate was cleared by centrifugation for 15 min at 13 500 rpm and 4 °C. The supernatant was then loaded onto a 10–50% sucrose gradient. After ultracentrifugation at 25 000 rpm and 4 °C for 4 h using Beckman LE‐80K, the gradients were fractionated into #1 to #12 (from light to heavy) fractions and analyzed for RNA contents in each fraction using the Gradient Station Base Unit (Biocomp). For RNA sequencing analysis of actively translated mRNAs, RNAs were isolated from the fraction #8 to #11 were using TRIzol and Glycogen (Nacalai Tesque).

### Xenograft Experiments and Limiting Dilution Assay

Female 6 to 8 week old BALB/c‐nu/nu immunocompromised mice (Japan SLC) were used for xenograft experiments. 72 h before transplantation, RMS‐YM cells were infected with Control‐shRNA‐, CDKAL1‐shRNA‐, or SALL2‐shRNA‐expressing lentivirus. One million cells were resuspended in 100 µL PBS containing 50% Matrigel (Corning) and were subcutaneously injected into each flank of the mouse (ten injection sites per five mice). One million cells of Control‐C2C12 or HRas/shp53‐C2C12 cells were injected into mice as same as above to evaluate the tumorigenic potential of HRas/shp53‐C2C12 cells. Tumor volume was determined by calculating the equation of 3.14 × *D* × *d* ^2^)/6 (“*D*” represents the maximum diameter of the tumor and “*d*” represents the minimum diameter of the tumor). HRas/shp53‐C2C12 cells were infected with Control‐shRNA‐ or CDKAL1‐shRNA‐ ‐expressing lentivirus for the limiting dilution assay. Each cell at three different concentrations (1 × 10^5^ cells/100 µL, 1 × 10^4^ cells/100 µL, and 1 × 10^3^ cells/100 µL) was subcutaneously injected into each flank (eight injection sites per four mice). Extreme Limiting Dilution Analysis (ELDA, http://bioinf.wehi.edu.au/software/elda/) was used to analyze the results of limiting dilution experiments. All animal experiments were carried out under permission from the animal ethics committee of Okayama University (approval number: OKU‐2019380).

### Immunostaining

24 h before immunostaining analysis, cells were seeded on 8‐well chamber slides (Thermo Fisher Scientific). The cells were fixed with 4% PFA for 20 min at room temperature, permeated with 0.05% PBST (PBS with 0.05% Triton X‐100) for 10 min, and blocked with 3% bovine serum albumin (BSA) (Sigma‐Aldrich) in PBST (BSA‐PBST) for 1 h at room temperature. The cells were incubated with primary antibodies in BSA‐PBST at 4 °C overnight. After a brief wash, the cells were then incubated with secondary antibodies in BSA‐PBST at room temperature for 2 h and mounted with DAPI‐Fluoromount‐G (SouthernBiotech). For immunostaining of frozen sections, tumors were excised and immediately fixed with 4% PFA for at least 24 h. The tumors were embedded in Tissue‐Tek O.C.T. Compound (Sakura Finetek) and sliced into 10 µm thick sections using a cryostat (Leica). The sections were incubated with HistoVT One (Nacalai Tesque) for antigen retrieval following the manufacturer's instructions and stained as same as the cells were stained. For immunostaining of the paraffin‐embedded RMS tissue array (US Biomax), the section was deparaffinized with xylene before the staining procedures as same as described above. The detailed information of the array is available on the website (https://www.biomax.us/tissue‐arrays/Soft_Tissue/SO2082b). All samples were observed using a confocal microscope LSM780 (Carl Zeiss AG) and analyzed with ZEN software (Carl Zeiss AG). We also used ImageJ software to quantify relative expression levels by measuring the mean gray value (Integrated Density/Area) of each sample. All antibodies used in the study are shown above.

### In Situ Hybridization and Proximity Ligation Assay

Digoxigenin (DIG)‐labeled RNA probes were prepared as previously described.^[^ ^49^ ^]^ Briefly, the sequence of SALL2 5’UTR or the corresponding inverse sequence was cloned into pEF1alpha‐IRES at the restriction enzyme site of NheI. The plasmids were then digested with EcoRI and gel‐purified. Antisense or sense probes were transcribed from the T7 promoter of the digested plasmids using a DIG RNA labeling kit (Sigma‐Aldrich). Before the in situ hybridization procedure, the cells seeded on eight‐well chamber slides were fixed with 4% PFA and permeabilized with 0.05% PBST, and the DIG‐labeled RNA probes were denatured at 90 °C for 10 min in hybridization buffer. The cells were incubated with hybridization buffer (50% Formamide (Sigma‐Aldrich), 2 × saline‐sodium citrate (SSC) (Thermo Fisher Scientific), 1% SDS) for 10 min at room temperature. The cells were incubated with the denatured probes at 37 °C overnight, and washed three times with hybridization buffer at 37 °C for 5 min, three times with 2 × SSC at 37 °C for 5 min, and once with 1 × SSC solution at 37 °C for 5 min. Then, the cells were washed twice with 4 × SSC solution at room temperature for 10 min and subjected to the proximity ligation assay (PLA). The PLA was performed using Duolink PLA reagents (Sigma‐Aldrich) according to the manufacturer's instructions. The cells were incubated with Duolink Blocking Solution for 1 h at 37 °C and primary antibodies overnight at 4 °C. After a brief wash with PBST, the cells were incubated with PLUS and MINUS PLA probes for 1 h at 37 °C, followed by incubation with ligation mix for 30 min at 37 °C. Amplification mix was then applied for 100 min at 37 °C. After mounting with DAPI‐Fluoromount‐G, the Duolink images were acquired by using a confocal microscope LSM780. All antibodies used in the study are shown above.

### Bioinformatics Analysis of Human RMS Datasets

The gene expression profile of RMS patients and normal muscle was obtained from public datasets (Gene Expression Omnibus (GEO): GSE108022). The dataset used for Kaplan‐Meier analysis and Gene Set Enrichment Analysis (GSEA) was collected from ArrayExpress (E‐TABM‐1202). For the survival curve analysis, the RMS patients were stratified by the mean value of CDKAL1 or SALL2. Based on the clinical information provided in the dataset, the survival curve analysis was done by using GraphPad Prism (V.8.0). Version 4.1.0 of the GSEA desktop application was used to perform GSEA. CDKAL1 expression was set into high and low categories based on the mean expression value. CDKAL1‐correlated gene sets were obtained by collapsing the data into gene symbols and running 1000 permutations using a weighted enrichment statistic. Gene sets were filtered for a minimum of 15, and a maximum of 500 genes and genes were ranked by signal to noise ratios for the curated gene sets (c2.all.v7.1.symbols.gmt). Gene sets with *P*  <  0.05 and false‐discovery rate (FDR)  <  0.25 were significantly enriched. The human protein atlas (https://www.proteinatlas.org) was used to obtain the survival information of other cancer types (melanoma, liver cancer, prostate cancer, and stomach cancer). The information related to glioma was obtained from The Cancer Genome Atlas (TCGA) dataset.

### Analysis of RNA‐Seq Data

RNA sequence data were obtained and processed by Rhelixa. RNA sequencing analyses were performed with total RNA and RNA from the actively translated polysome fractions in RD cells expressing Control‐shRNA, CDKAL1‐shRNA#1, and CDKAL1‐shRNA#2, with two replicates per each condition (total RNA‐shControl‐1, total RNA‐shControl‐2, total RNA‐shCDKAL1#1‐1, total RNA‐shCDKAL1#1‐2, total RNA‐shCDKAL1#2‐1, total RNA‐shCDKAL1#2‐2, polysome RNA‐shControl‐1, polysome RNA‐shControl‐2, polysome RNA‐shCDKAL1#1‐1, polysome RNA‐shCDKAL1#1‐2, polysome RNA‐shCDKAL1#2‐1, and polysome RNA‐shCDKAL1#2‐2). Transcripts per million value was used for the analyses. We filtered low expressing genes (the value was less than 20 in total RNA‐shControl condition) from the raw counts matrix. The genes whose expression levels in total RNA were altered upon CDKAL1 knockdown were considered transcriptionally changed genes, and those in polysome RNA were considered translationally changed genes. CDKAL1‐dependent genes (genes whose expression levels were translationally changed upon CDKAL1 knockdown) were defined according to the differentially expressed genes with fold change −0.5 < log2 < 0.5 in total RNA condition and log2 < −0.5 in polysome RNA condition. The coding sequence (CDS) and 5’UTRs were obtained from the UCSC Genome Browser on Human (GRCh38/hg38). RefSeq annotated mRNAs with known CDSs and 5’UTRs were collected for further analysis. The CDS of CDKAL1‐dependent genes and all the other were put separately into Sequence Manipulation Suite (Codon Usage) (https://www.bioinformatics.org/sms2/codon_usage.html) and the lysine codon frequency (AAA and AAG) was determined. The 5’UTR sequence of CDKAL1‐dependent genes were put into Multiple Em for Motif Elicitation (MEME 5.1.1), and the CES and GES motifs were identified using search parameters for a 6‐50 nucleotide sequence with any number of repeats. Then the CDKAL1‐dependent genes whose 5’UTR containing CES or GES were analyzed by g:Profiler (gene ID conversion and functional profiling) (https://biit.cs.ut.ee/gprofiler/gost). RNA sequence data were deposited to GEO.

### Fluorescence‐Activated Cell Sorting

RD and RH30 cells were stained with Brilliant Violet 421‐labeled antibody against CD133 (BioLegend) for 30 min on ice to isolate CD133^high^ and CD133^low^ populations. The cells were washed with FACS buffer (0.12% BSA in PBS) three times. Then, the cells were incubated with FACS buffer containing 1 µg mL^‐1^ propidium iodide (Nacalai Tesque) for 30 min to stain dead cells. The Brilliant Violet 421‐labeled mouse IgG1, *κ* (BioLegend) was set as the isotype control. The profile analysis and cells sorting were done using FlowJo 10.4 and BD FACSAria III Cell Sorter (BD Biosciences). Sorted RD cells were used for sphere‐formation assay, colony formation assay, protein extraction, RNA isolation, and immunofluorescent analysis.

### Analysis of Cap‐Bound Proteins

7‐methyl‐guanosine‐5’‐triphosphate (m^7^GTP)‐sepharose beads (Jena Bioscience) were used to capture eIF4E and its binding proteins. For pull‐down experiments, cells were seeded on 10 cm culture dish and lysed with 1 mL of lysis buffer supplemented with 0.5% NP‐40. After brief destruction of cells with a 23G needle syringe, the lysate was cleared by centrifugation at 13 500 rpm and 4 °C for 15 min. The supernatant was then subjected to incubation with m^7^GTP beads for 4 h at 4 °C. After washing with lysis buffer with 0.5% NP‐40, the samples were boiled in 2× SDS sample buffer at 95 °C for 5 min and subjected to western blotting analyses.

### Luciferase Assay

For luciferase experiments, the coding sequence of firefly luciferase was first cloned into pEF1alpha‐IRES (Clontech) at the restriction enzyme site of NheI/MluI. Then, the 5’UTR sequences of SALL2, GAPDH, and ACTB mRNAs were cloned into pEF1alpha‐firefly luciferase‐IRES at the restriction enzyme site of NheI. RD cells expressing Control‐shRNA or CDKAL1‐shRNA were cotransfected with 5’UTR‐firefly luciferase reporter and Renilla luciferase reporter pGL4.74[hRluc/TK] (Promega) at a ratio of 3:2 using TransIT‐LT1 and Opti‐MEM. 24 h after transfection, the cells were harvested, and the luciferase activities were measured using a Dual‐Luciferase Reporter Assay System (Promega). The firefly luciferase activity was normalized to Renilla luciferase activity and showed as values relative to the signals obtained from RD cells expressing Control‐shRNA.

### Cell Proliferation Assay

Cell proliferation assay was performed by using CellTiter 96 AQueous One Solution Cell Proliferation Assay kit according to the manufacturer's instructions (Promega). Two days before the assay, RD or RH30 cells with Control‐shRNA‐ or CDKAL1‐shRNA‐expressing lentivirus were infected. On the day before the measurement (day 0), one thousand of the infected cells were seeded to the wells of a 96‐well plate in DMEM with 10% FBS and 1% antibiotics. On each day of measurement, 20 µL per well of the reagent was added. After 1 h incubation at 37 °C in a cell culture chamber, the absorbance at 490 nm was recorded using a plate reader.

### RNA Immunoprecipitation and Reverse Transcription PCR

An RNA immunoprecipitation and reverse transcription PCR was performed as described with a modification.^[^ ^50^ ^]^ Briefly, RD cells expressing myc‐tagged CDKAL1^WT^ were grown at 80% confluency on a 10 cm cell culture dish and subjected to cross‐linking by addition of formaldehyde (0.75% final concentration) for 10 min at room temperature. Cross‐linking was terminated with 125 × 10^‐3^ m glycine for 10 min at room temperature. The cells were then lysed in lysis buffer containing 100 U RNaseOUT recombinant ribonuclease inhibitor (Thermo Fisher). Lysate was cleared by centrifugation at 10 000 g for 10 min at 4 °C and the supernatant was then subjected to the incubation with 50 µL of anti‐myc‐tag mAb‐Magnetic Beads (MBL Life Science) for 16 h at 4 °C. After brief wash with lysis buffer, the samples were incubated at 65 °C for 2 h in reverse cross‐linking buffer (50 × 10^‐3^ m Tris‐HCl (pH = 7.5), 5 × 10^‐3^ m EDTA, 10 × 10^‐3^ m DTT, 10 U RNaseOUT), and RNA was extracted by using the TRIzol reagent. The complementary DNA was synthesized as described above. PCR was performed by using a KOD‐FX Neo kit (Toyobo), and the samples were run on 1.2% agarose gel and stained with ethidium bromide.

### Statistical Analyses

The number of replicates and animals are indicated in figure legends or Experimental Section. The statistical analyses were done using GraphPad Prism (V.8.0), and the results were presented as the mean ± SD. An unpaired two‐tailed t‐test was used to calculate the significant differences of each comparison. One‐way ANOVA with Tukey's multiple comparisons test was used for more than two groups. The log‐rank (Mantel‐Cox) test was applied for the survival curve analysis to find the statistical significance between survival curves. A *P*‐value less than 0.05 was considered to be statistically significant. The significance level was defined as ns (no significance), *P < 0.05; **P < 0.01; ***P < 0.001; ****P < 0.0001.

## Conflict of Interest

Atsushi Fujimura is applying a patent that might be connected to the manuscript. The other authors declare that they have no competing interests.

## Author Contributions

R.S.H. performed most of the experiments in vitro and in vivo, carried out bioinformatics analyses, and contributed to the writing. T.Y. carried out the initial experiments of this study using glioma cells. E.N., T.O., K.K., F.Y.W., and K.T. reviewed and edited the manuscript. A.F. conceived the initial hypothesis and experimental design, acquired funding, organized the work and wrote the manuscript.

## Supporting information

Supporting Information

## Data Availability

RNA sequence data will be deposited to GEO.
