# Supplementary material for: CDKAL1 Drives the Maintenance of Cancer Stem‐Like Cells by Assembling the eIF4F Translation Initiation Complex
Source: Adv Sci (Weinh). 2023 Feb 14;10(12):2206542. doi: 10.1002/advs.202206542 (PMC10131790; doi:10.1002/advs.202206542)
Supplement: Supplementary file 1 — Supporting Information [file ADVS-10-2206542-s001.pdf]

ADVANCED SCIENCE
Open Access

Supporting Information

for *Adv. Sci.*, DOI 10.1002/advs.202206542

CDKAL1 Drives the Maintenance of Cancer Stem-Like Cells by Assembling the eIF4F Translation Initiation Complex

*Rongsheng Huang, Takahiro Yamamoto, Eiji Nakata, Toshifumi Ozaki, Kazuhiko Kurozumi, Fanyan Wei, Kazuhito Tomizawa and Atsushi Fujimura**

Supporting Information

Title: CDKAL1 drives the maintenance of cancer stem-like cells by assembling the eIF4F translation initiation complex

Rongsheng Huang[1], *Takahiro Yamamoto*[2], *Eiji Nakata*[3], *Toshifumi Ozaki*[3], *Kazuhiko Kurozumi*[4], *Fanyan Wei*[5], *Kazuhito Tomizawa*[2], *and Atsushi Fujimura*[1,6*]

[1] Department of Cellular Physiology, Okayama University Graduate School of Medicine, Dentistry, and Pharmaceutical Sciences, Okayama, Japan

[2] Department of Molecular Physiology, Kumamoto University Faculty of Life Sciences, Kumamoto, Japan

[3] Department of Orthopedic Surgery, Okayama University Graduate School of Medicine, Dentistry, and Pharmaceutical Sciences, Okayama, Japan

[4] Department of Neurosurgery, Hamamatsu University School of Medicine, Hamamatsu, Japan

[5] Department of Modomics Biology and Medicine, Institute of Development, Aging and Cancer, Tohoku University, Sendai, Japan

[6]Neutron Therapy Research Center, Okayama University, Okayama, Japan

[*] To whom correspondence should be addressed

Atsushi Fujimura

Department of Cellular Physiology, Okayama University Graduate School of Medicine, Dentistry, and Pharmaceutical Sciences

2-5-1 Shikata-cho, kita-ku, Okayama, 700-8558, Japan

TEL: +81-86-235-7105

FAX: +81-86-235-7111

Email Address: atsushi.fujimura@okayama-u.ac.jp

Figure S1. CDKAL1 promotes the maintenance of melanoma CSC-related traits.

(**A**) The prognosis of CDKAL1^{high} patients was significantly worse than that of the CDKAL1^{low} patients in melanoma.

(**B**) The self-renewal capacity of Control-shRNA- or *CDKAL1*-shRNA-expressing human melanoma cell lines, A2058, SK-Mel-28, and HMV-II ($n = 4$, error bars indicate mean ± SD). Western blotting results are shown to confirm *CDKAL1* knockdown.

(**C**) Representative images of the clonogenic assay in Control-shRNA- or *CDKAL1*-shRNA-expressing human melanoma cells.

(**D**) Immunofluorescent analysis using antibodies against melanoma CSC-markers, ALDH1 or CD44, in Control-shRNA- or *CDKAL1*-shRNA-expressing human melanoma cells. Scale bars, 50 μm.

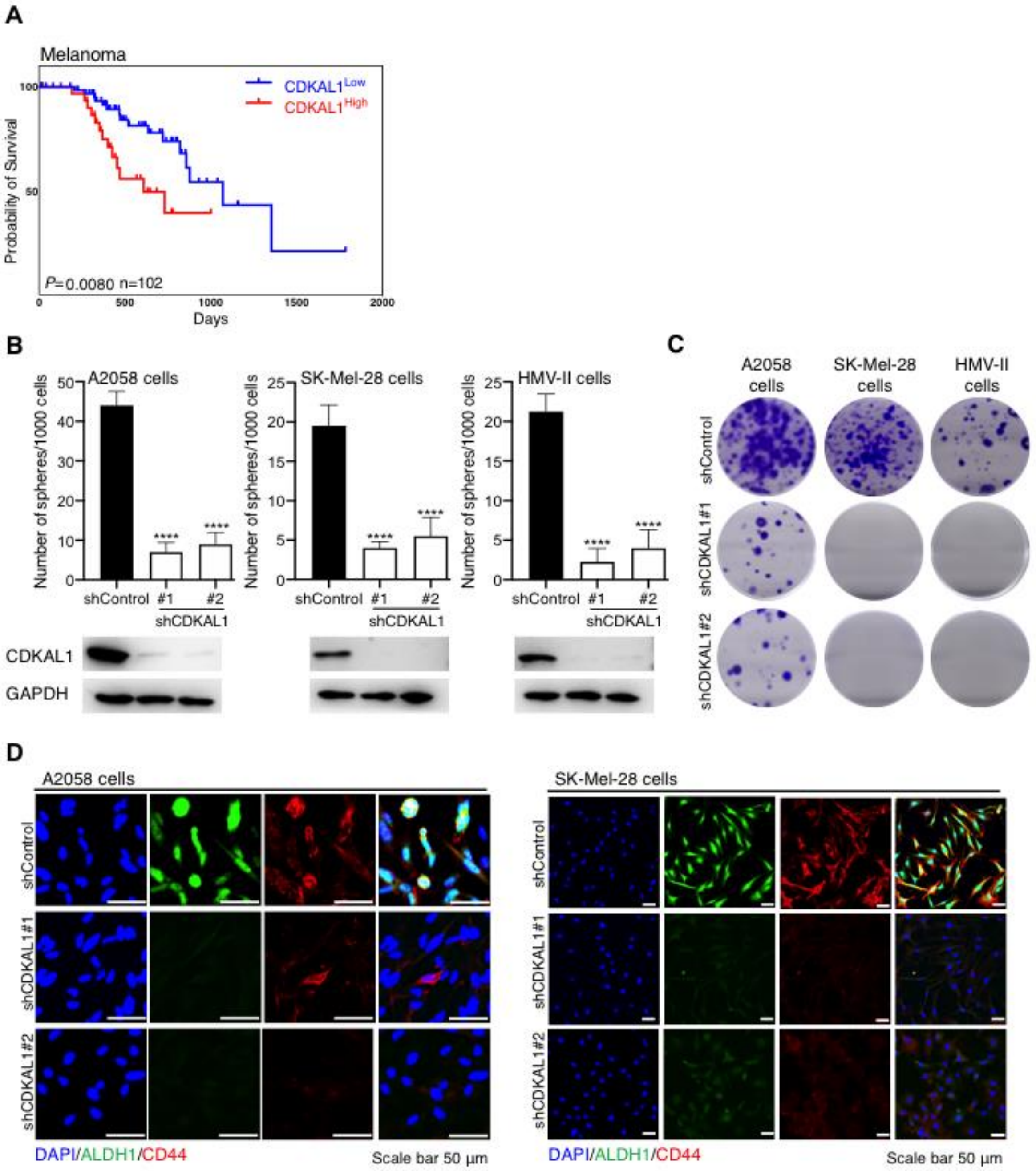

Figure S1 Huang et al.

Figure S2. CDKAL1 promotes the maintenance of liver cancer CSC-related traits.

(**A**) The prognosis of $CDKAL1^{high}$ patients was significantly worse than that of the $CDKAL1^{low}$ patients in liver cancer.

(**B**) The self-renewal capacity of Control-shRNA- or *CDKAL1*-shRNA-expressing human liver cancer cell lines, HuH-7 and HepG2 ($n = 4$, error bars indicate mean ± SD). Western blotting results are shown to confirm *CDKAL1* knockdown.

(**C**) Representative images of the clonogenic assay in Control-shRNA- or *CDKAL1*-shRNA-expressing human liver cancer HepG2 cells.

(**D**) Immunofluorescent analysis using antibodies against liver cancer CSC-markers, CD133 or CD44, in Control-shRNA- or *CDKAL1*-shRNA-expressing human liver cancer cells. Scale bars, 50 μm.

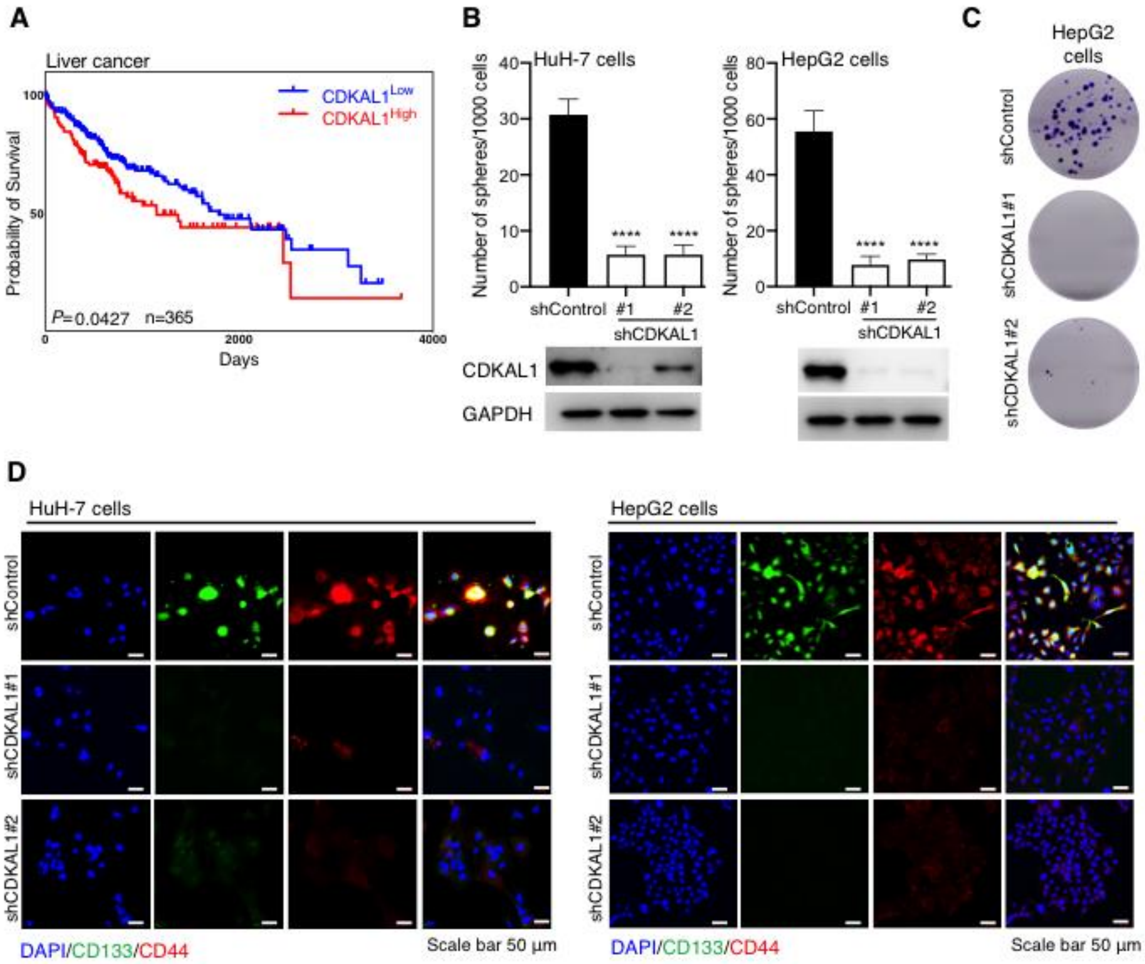

Figure S2 Huang et al.

Figure S3. CDKAL1 promotes the maintenance of prostate cancer CSC-related traits.

(**A**) The prognosis of CDKAL1^{high} patients was significantly worse than that of the CDKAL1^{low} patients in prostate cancer.

(**B**) The self-renewal capacity of Control-shRNA- or *CDKAL1*-shRNA-expressing human prostate cancer cell lines, PC3 and LNCaP ($n = 4$, error bars indicate mean ± SD). Western blotting results are shown to confirm *CDKAL1* knockdown.

(**C**) Immunofluorescent analysis using antibodies against prostate cancer CSC-markers, CD133 or CD44, in Control-shRNA- or *CDKAL1*-shRNA-expressing human prostate cancer cells. Scale bars, 50 μm.

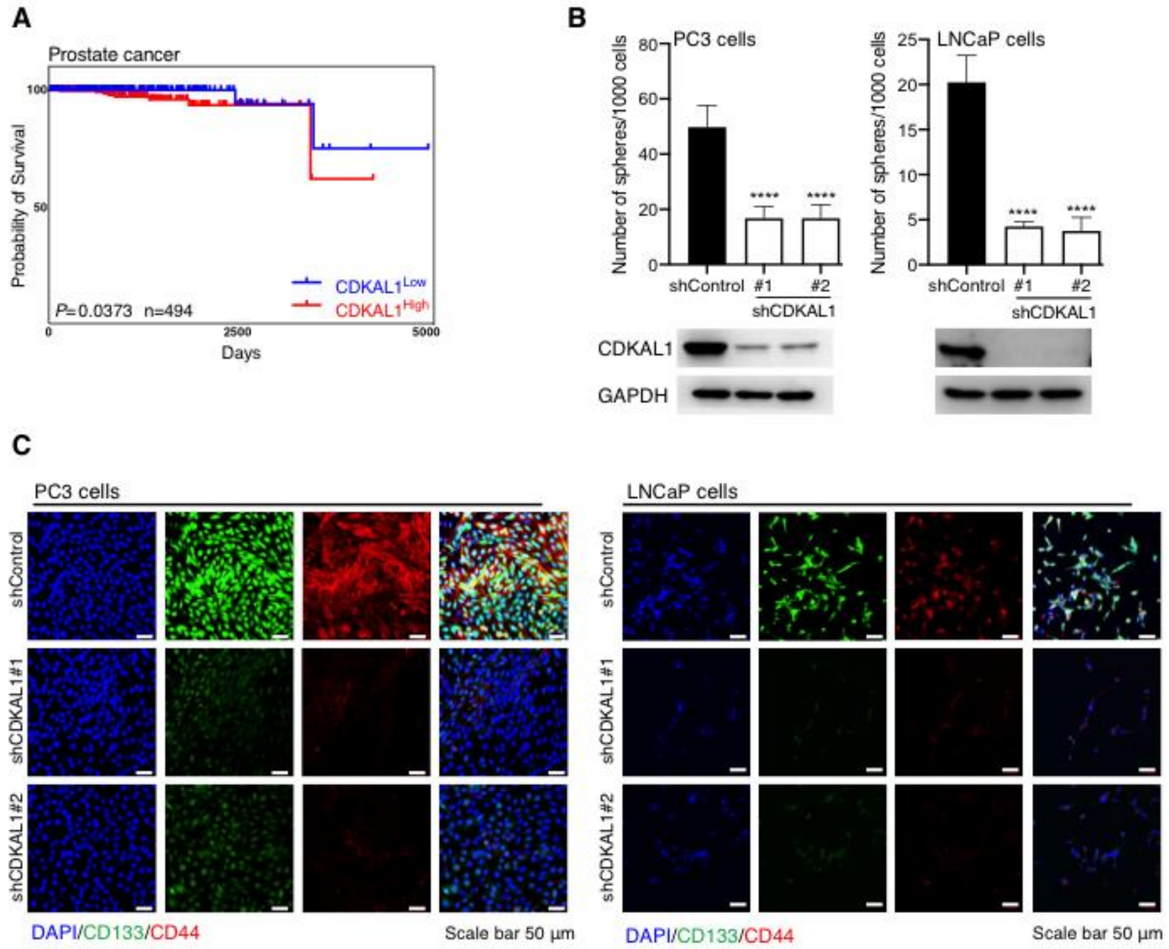

Figure S3 Huang et al.

Figure S4. CDKAL1 promotes the maintenance of stomach cancer CSC-related traits.
(**A**) The prognosis of CDKAL1^{high} patients was significantly worse than that of the CDKAL1^{low} patients in stomach cancer.
(**B**) The self-renewal capacity of Control-shRNA- or *CDKAL1*-shRNA-expressing human stomach cancer cell lines, NUGC3, HGC27, and MKN45 ($n = 4$, error bars indicate mean ± SD). Western blotting results are to confirm *CDKAL1* knockdown.
(**C**) Representative images of the clonogenic assay in Control-shRNA- or *CDKAL1*-shRNA-expressing human stomach cancer NUGC3 and MKN45 cells.
(**D**) Immunofluorescent analysis using antibodies against stomach cancer CSC-markers, ALDH1 or CD44, in Control-shRNA- or *CDKAL1*-shRNA-expressing human stomach cancer cells. Scale bars, 50 μm.

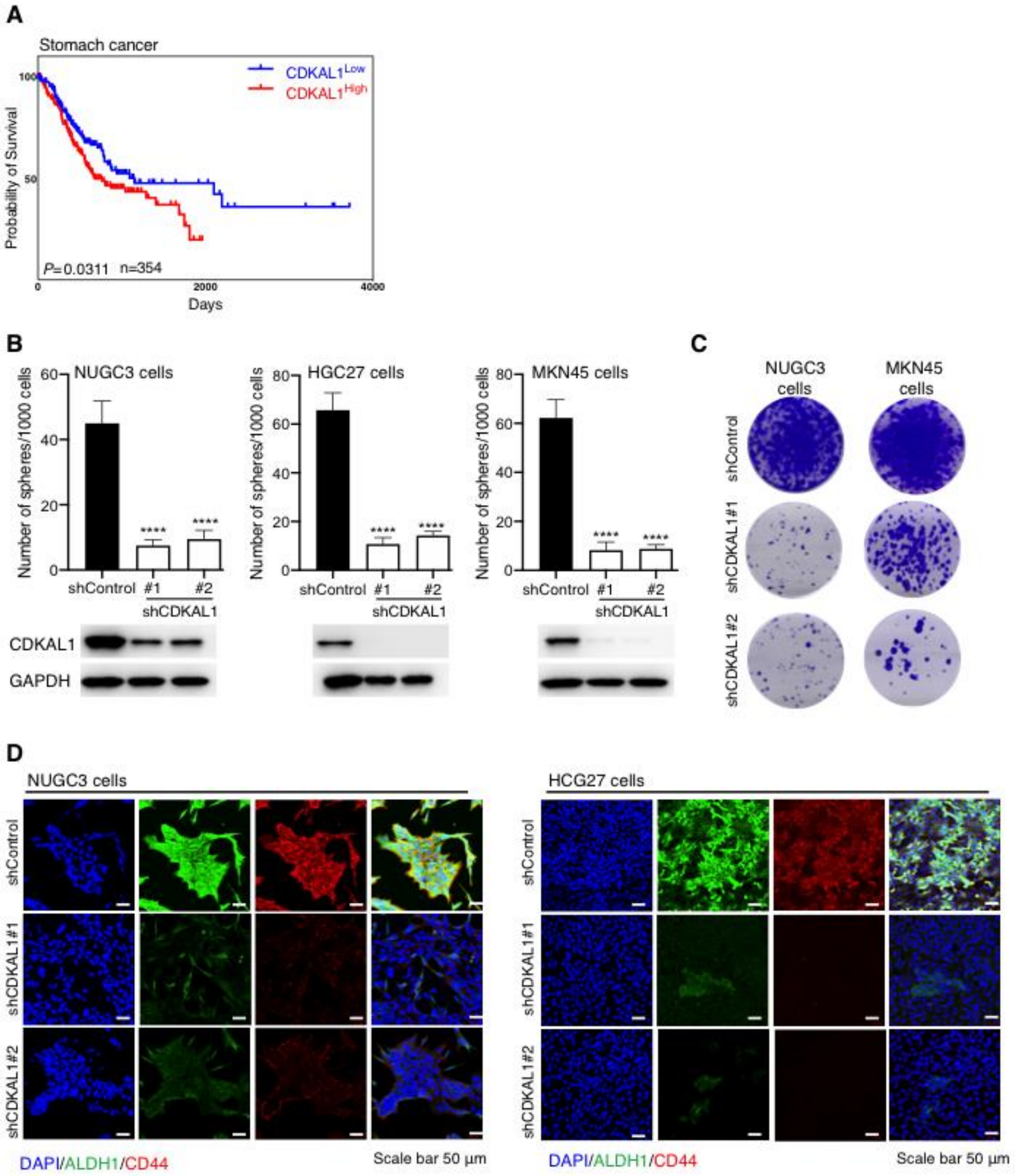

Figure S4 Huang et al.

Figure S5. CDKAL1 promotes the maintenance of glioma CSC-related traits.

(**A**) The prognosis of $CDKAL1^{high}$ patients was significantly worse than that of the $CDKAL1^{low}$ patients in glioma.

(**B**) Western blotting analysis of glioma CSC-related factors, SOX2, POU3F2, OLIG2, and CD44, in Control-shRNA- or *CDKAL1*-shRNA-expressing human glioma cell lines, MGG4, MGG8, and MGG18.

(**C**) Results of single-cell sphere-formation assay in Control-shRNA- or *CDKAL1*-shRNA-expressing human glioma cell lines, MGG4, MGG8, and MGG18 ($n = 3$, error bars indicate mean ± SD).

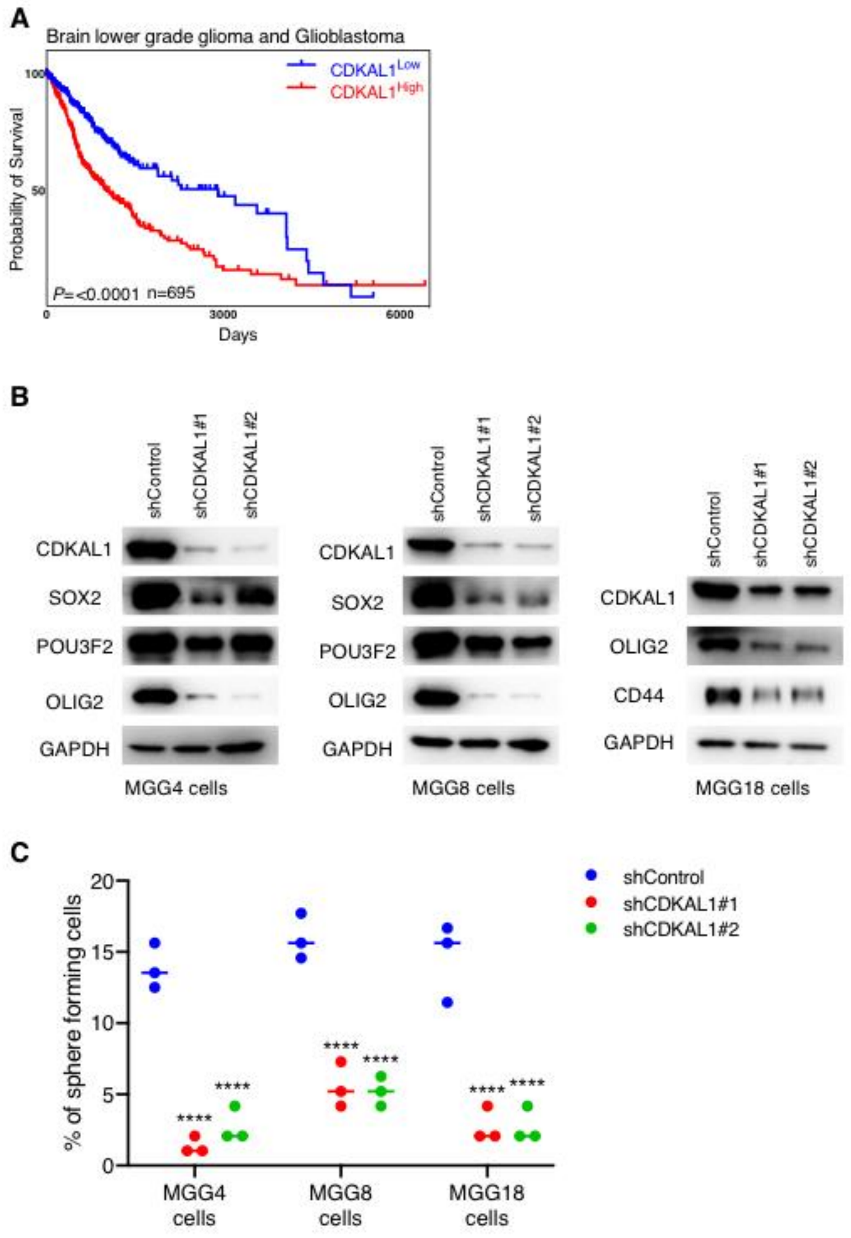

Figure S5 Huang et al.

Figure S6. SALL2 is a CSC-related transcription factor in melanoma.

(**A**) Immunofluorescent analysis using anti-SALL2, anti-CDKAL1, and anti-CD44 antibodies in the human melanoma cell line A2058. Scale bars, 50 μm.

(**B**) Western analysis showing the effect of *CDKAL1* knockdown on SALL2 expression levels in human melanoma cell lines A2058, SK-Mel-28, and HMV-II.

(**C**) The self-renewal capacity of Control-shRNA- or *SALL2*-shRNA-expressing human melanoma cell lines ($n = 4$, error bars indicate mean ± SD). Western blotting results are shown to confirm *SALL2* knockdown.

(**D**) Representative images of the clonogenic assay in Control-shRNA- or *SALL2*-shRNA-expressing human melanoma cells.

(**E**) Immunofluorescent analysis using antibodies against melanoma CSC-markers, ALDH1 and CD44, in Control-shRNA- or *SALL2*-shRNA-expressing human melanoma cells. Scale bars, 50 μm.

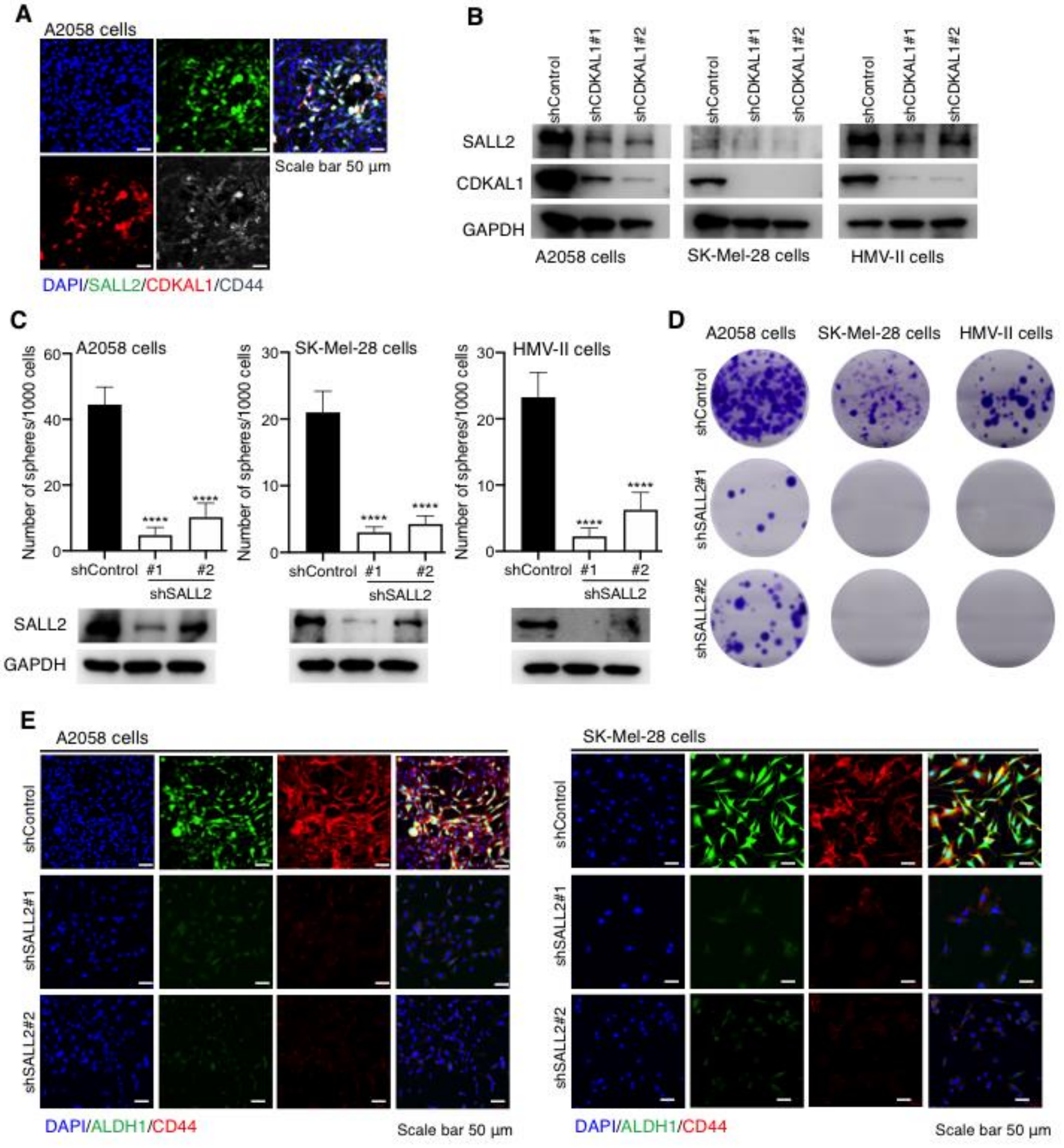

Figure S6 Huang et al.

Figure S7. SALL2 is a CSC-related transcription factor in liver cancer.

(**A**) Immunofluorescent analysis using anti-SALL2, anti-CDKAL1, and anti-CD44 antibodies in the human liver cancer cell line HepG2. Scale bars, 50 μm.

(**B**) Western analysis showing the effect of *CDKAL1* knockdown on the expression levels of SALL2 in human liver cancer cell lines HuH-7 and HepG2.

(**C**) The self-renewal capacity of Control-shRNA- or *SALL2*-shRNA-expressing human liver cancer cell lines ($n = 4$, error bars indicate mean ± SD). Western blotting results are shown to confirm *SALL2* knockdown.

(**D**) Representative images of the clonogenic assay in Control-shRNA- or *SALL2*-shRNA-expressing HepG2 cells.

(**E**) Immunofluorescent analysis using antibodies against liver cancer CSC-markers, CD133 and CD44, in Control-shRNA- or *SALL2*-shRNA-expressing human liver cancer cells. Scale bars, 50 μm.

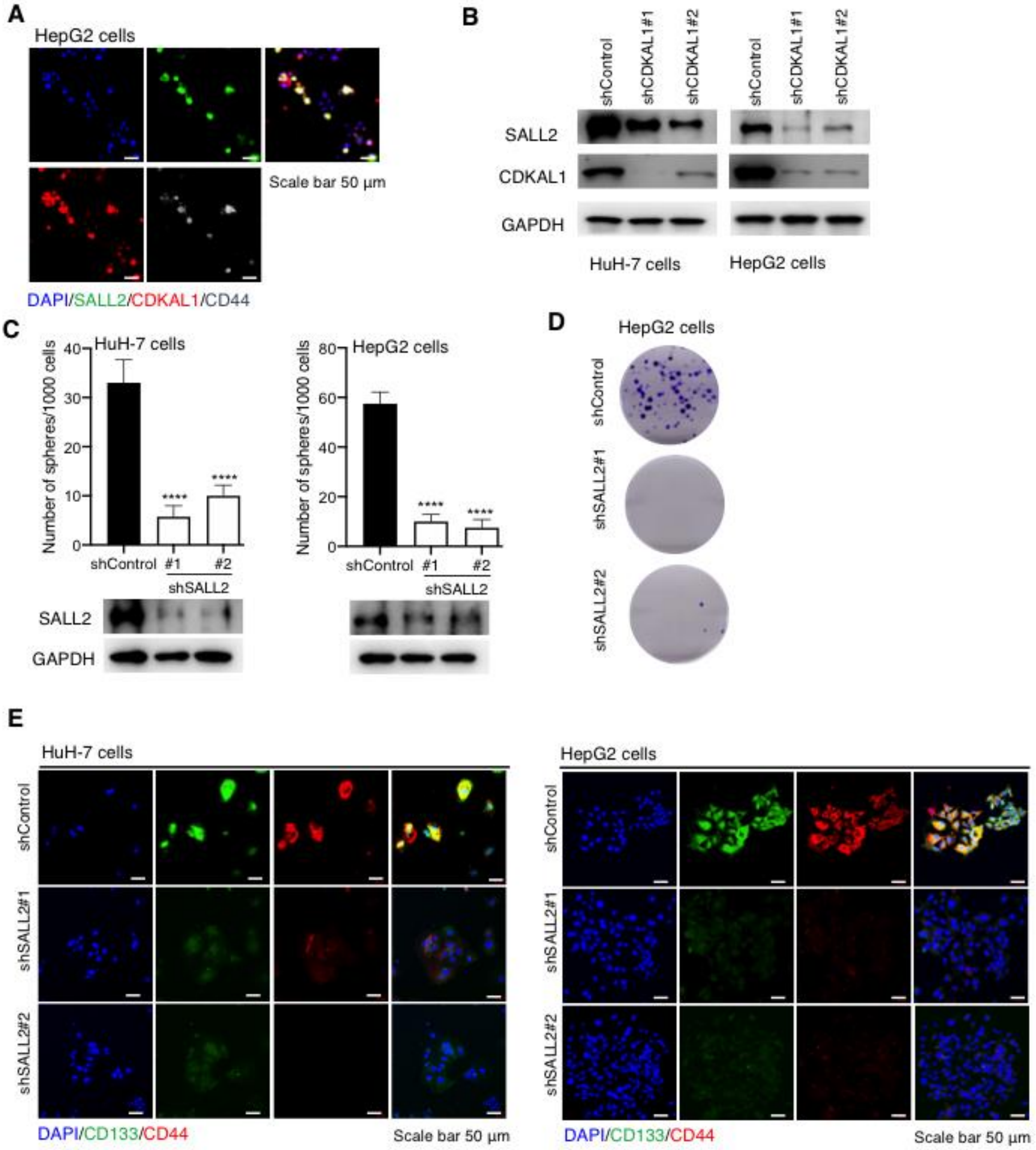

Figure S7 Huang et al.

Figure S8. SALL2 is a CSC-related transcription factor in prostate cancer.

(**A**) Immunofluorescent analysis using anti-SALL2, anti-CDKAL1, and anti-CD44 antibodies in the human prostate cancer cell line PC3. Scale bars, 50 μm.

(**B**) Western analysis showing the effect of *CDKAL1* knockdown on SALL2 expression levels in human prostate cancer cell lines PC3 and LNCaP.

(**C**) The self-renewal capacity of Control-shRNA- or *SALL2*-shRNA-expressing human prostate cancer cell lines ($n = 4$, error bars indicate mean ± SD). Western blotting results are shown to confirm *SALL2* knockdown.

(**D**) Immunofluorescent analysis using antibodies against prostate cancer CSC-markers, CD133 and CD44, in Control-shRNA- or *SALL2*-shRNA-expressing human prostate cancer cells. Scale bars, 50 μm.

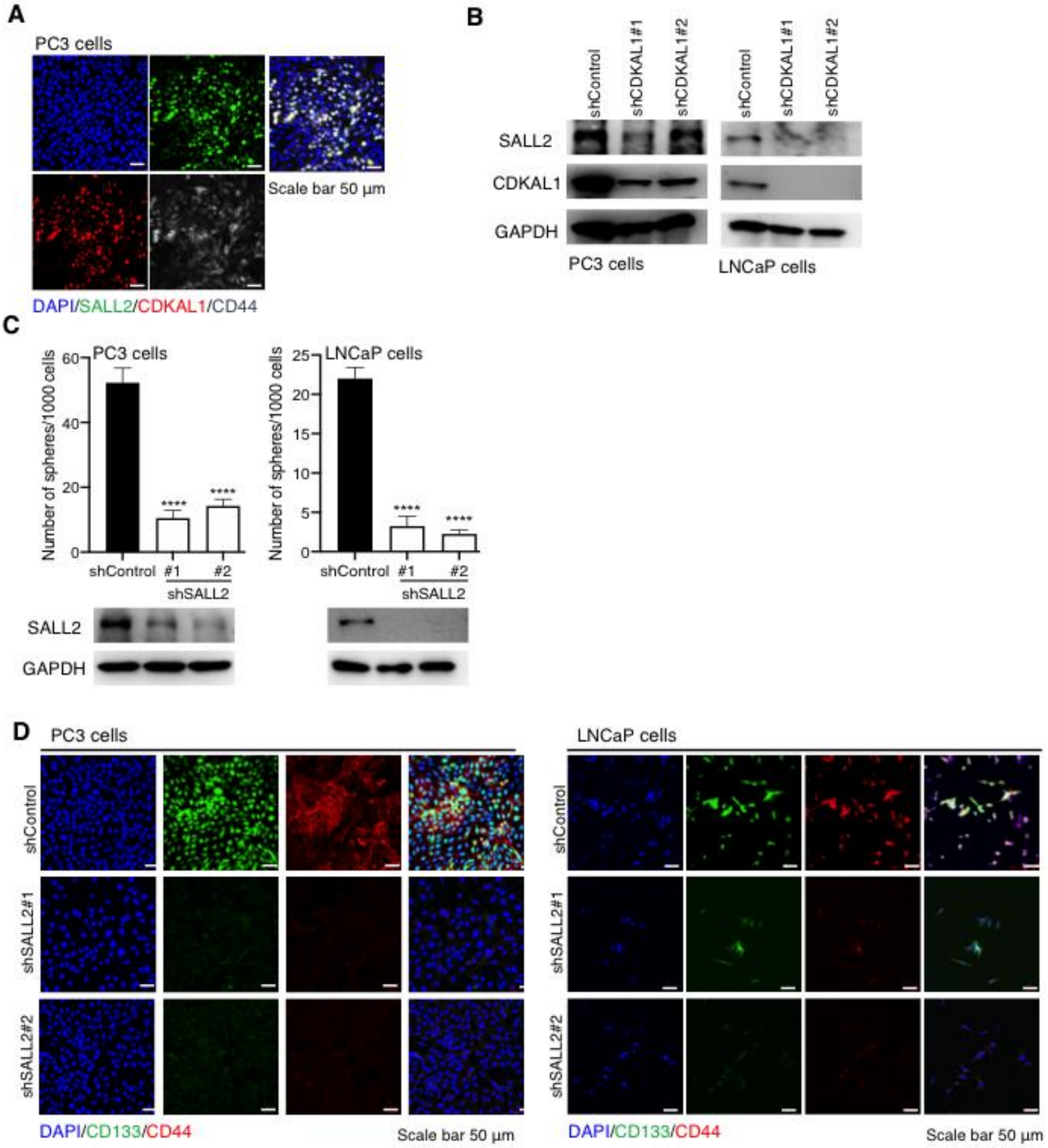

Figure S8 Huang et al.

Figure S9. SALL2 is a CSC-related transcription factor in stomach cancer.

(**A**) Immunofluorescent analysis using anti-SALL2, anti-CDKAL1, and anti-CD44 antibodies in human stomach cancer cell line NUGC3. Scale bars, 50 μm.

(**B**) Western analysis showing the effect of *CDKAL1* knockdown on the expression levels of SALL2 in human stomach cancer cell lines NUGC3, HGC27, and MKN45.

(**C**) The self-renewal capacity of Control-shRNA- or *SALL2*-shRNA-expressing human stomach cancer cell lines ($n = 4$, error bars indicate mean ± SD). Western blotting results are shown to confirm *SALL2* knockdown.

(**D**) Representative images of the clonogenic assay in Control-shRNA- or *SALL2*-shRNA-expressing NUGC3 and MKN45 cells.

(**E**) Immunofluorescent analysis using antibodies against stomach cancer CSC-markers, ALDH1 and CD44, in Control-shRNA- or *SALL2*-shRNA-expressing human stomach cancer cells. Scale bars, 50 μm.

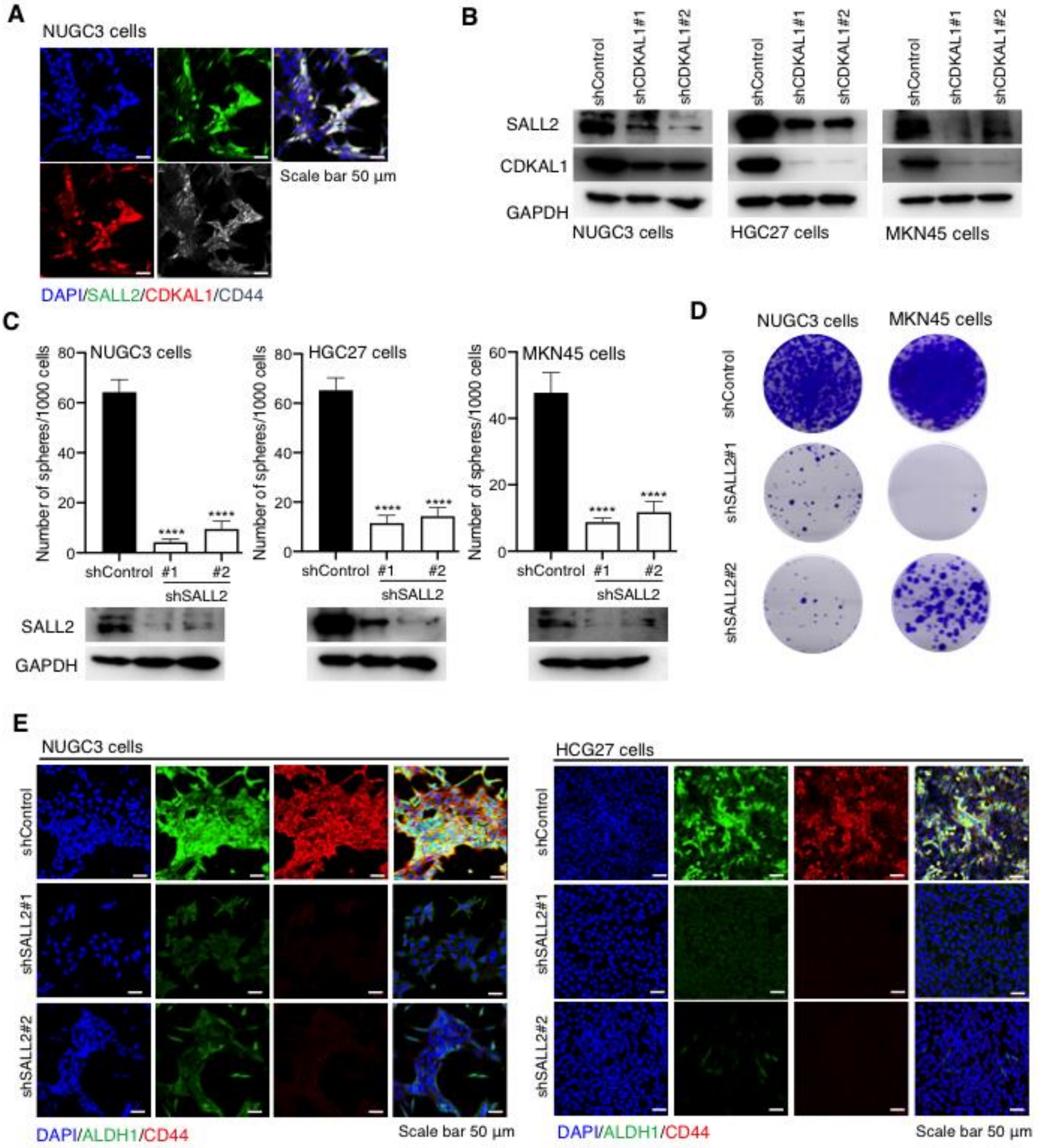

Figure S9 Huang et al.

Figure S10. SALL2 is a CSC-related transcription factor and CDKAL1 is required for promoting the assembly of the eIF4F translation initiation complex in glioma.
(**A**) Western blotting analysis showing the effect of *CDKAL1* knockdown on the expression levels of SALL2 in human glioma cell lines, MGG4, MGG8, and MGG18.
(**B and C**) Results of single-cell sphere-formation assay in Control-shRNA- or *SALL2*-shRNA-expressing human glioma cell lines, MGG4, MGG8, and MGG18 ($n = 3$, error bars indicate mean ± SD). Western blotting results are shown to confirm *SALL2* knockdown (**B**).
(**D**) Western blotting analysis of m^7GTP precipitate from Control-shRNA- or *CDKAL1*-shRNA-expressing human glioma cell lines MGG8 and MGG18.

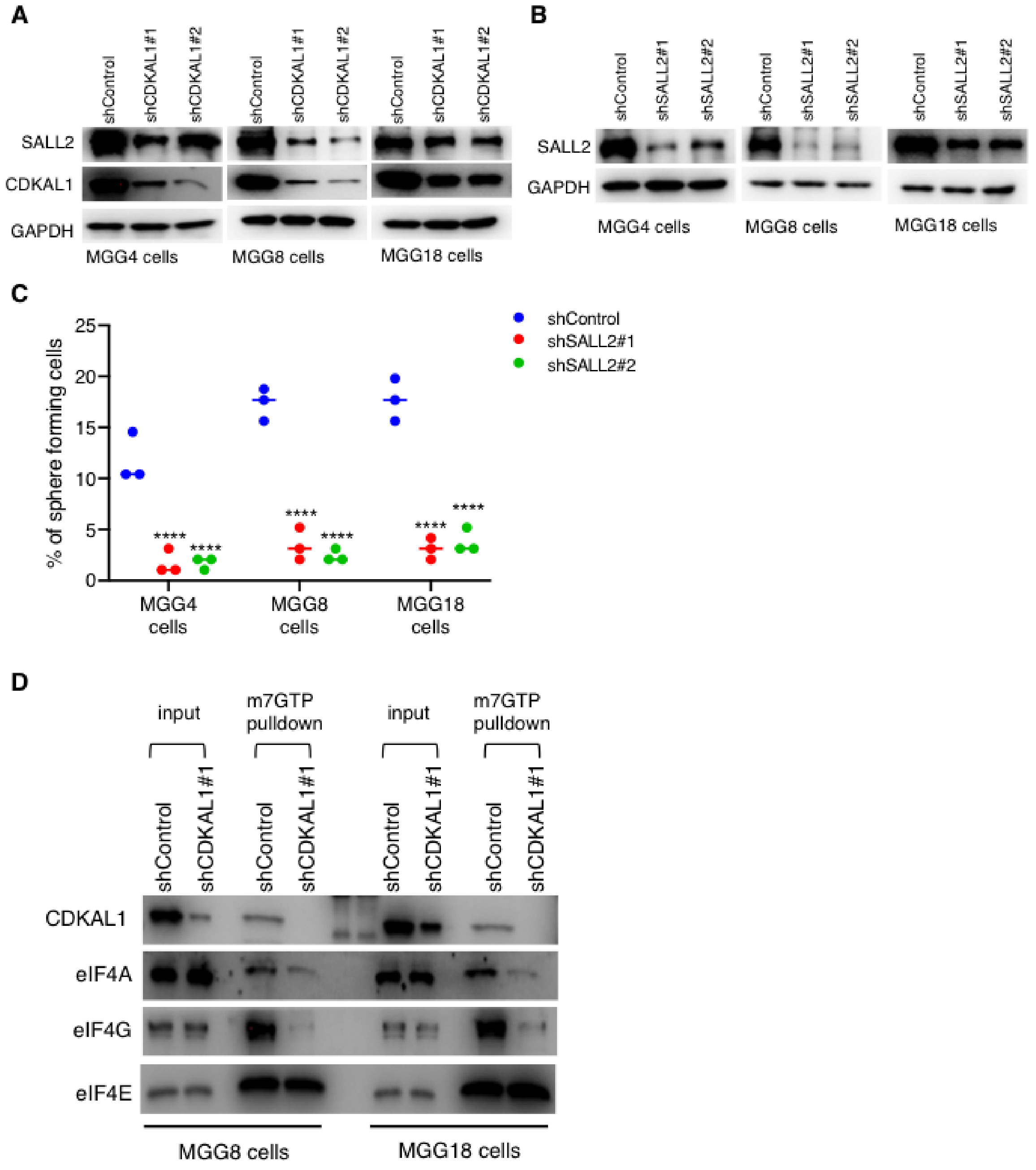

Figure S10 Huang et al.
